# Site-Specific Protein Bioconjugation Through Cellular Incorporation of Noncanonical Amino Acids

**DOI:** 10.1002/anie.1922738

**Published:** 2026-09-24

**Authors:** Rahul Sarkar, Manas Joshi, Abhishek Chatterjee

**Affiliations:** Department of Chemistry, Boston College, Chestnut Hill, Massachusetts, USA

**Keywords:** bioorthogonal chemistry, genetic code expansion, noncanonical amino acids, protein engineering, site-specific bioconjugation

## Abstract

Traditional methods for protein bioconjugation, such as those targeting canonical amino acid residues, often suffer from a lack of site-specificity, yielding heterogeneous conjugates. Genetic code expansion (GCE) technology offers an exciting alternative, enabling site-specific incorporation of noncanonical amino acids (ncAAs) bearing uniquely reactive chemical groups into recombinant proteins expressed in diverse host cells. The resulting proteins can be chemoselectively labeled at the installed ncAA residues under mild conditions to generate homogeneous, site-specific conjugates. In addition to offering site-specificity, the small size of the ncAA residues minimizes the risk of structural perturbation, allowing access to many more internal sites in proteins relative to alternative site-specific protein labeling approaches that require genetically encoded peptide and protein tags. Remarkable progress has been made in recent years, from improving the efficiency of ncAA incorporation at single or multiple sites, to expanding the palette of available chemistries, and applying this technology for both scientific discovery and the development of potential therapeutics and diagnostics. This review examines these advances and provides our perspectives on current challenges and future opportunities.

## 1 | Introduction

Protein bioconjugation is a powerful technology for both basic science and biotechnology applications [1–16]. Traditional protein bioconjugation strategies have primarily relied on the intrinsic reactivities of natural amino acids, such as lysine, cysteine, and tyrosine side chains, or the N-terminal amine [1–19]. However, these approaches frequently suffer from the inability to precisely control the site and stoichiometry of protein labeling (Figure 1A).

The need to generate homogeneous conjugates that preserve structure, activity, and reproducibility [20–22] has driven the development of alternative site-specific bioconjugation strategies. These include fusion of self-labeling peptide tags (e.g., tetracysteine and tetraserine tags) [23, 24], protein tags (e.g., SNAP-tag, CLIP-tag, HaloTag, and TMP-tag) [25–28], and enzyme-mediated labeling of peptide tags (e.g., biotin ligase and lipoic acid ligase) (Figure 1B) [29, 30]. Despite their utility, the considerable size of these tags often restricts where and how modifications can be introduced on a protein.

Over the last two decades, genetic code expansion (GCE) technology has emerged as a compelling alternative by enabling site-specific incorporation of noncanonical amino acids (ncAAs) with uniquely reactive chemical functionalities into proteins expressed in living cells [8, 9, 20, 31–40]. The ncAA side chains can be functionalized post-translationally with high selectivity and efficiency, resulting in homogeneous conjugates with precisely defined sites and stoichiometry of labeling (Figure 1C).

This technology provides distinct advantages over existing approaches. First, the site(s) of ncAA incorporation can be precisely defined by introducing a unique codon (e.g., the UAG stop codon) at the desired position(s) in the gene of interest. Second, ncAAs minimally perturb protein structure due to their small size, in contrast to bulky protein and peptide tags. This allows ncAA incorporation to be tolerated at many more internal positions, providing unprecedented flexibility in choosing labeling sites. Third, the development of mutually orthogonal bioorthogonal chemistries enables protein labeling with multiple distinct entities, either using a single ncAA bearing multiple reactive groups, or through incorporation of distinct ncAAs at different positions.

In this review, we summarize the principles underlying ncAA incorporation via GCE, examine the diverse chemistries employed for site-specific protein labeling using this approach, and highlight key applications it has enabled. We also discuss current challenges and future opportunities for expanding the scope and utility of GCE-mediated protein modification.

## 2 | Principles of Genetic Code Expansion

Site-specific incorporation of ncAAs requires reprogramming cellular translation using engineered translational components (Figure 2A) [31–34, 40, 41]. First, a unique codon specifies the incorporation site(s) in the target gene. While the repurposed UAG stop codon is commonly used, alternative approaches include other stop codons [42–48], quadruplet codons [49–54], rare sense codons [55–57], codons composed of nonnatural base pairs [58, 59], and liberated sense codons in engineered host cells with a compressed genetic code [60]. Second, an engineered aminoacyl-tRNA synthetase (aaRS)/tRNA pair delivers the ncAA in response to the unique codon. To preserve translational fidelity, this engineered pair must not cross-react with endogenous aaRS/tRNA systems (i.e., orthogonal).

Orthogonal pairs are typically borrowed from a different domain of life and subsequently engineered for ncAA incorporation (Figure 2B). The *M. jannaschii* tyrosyl-tRNA synthetase (MjTyrRS)/tRNA pair was among the first developed and has been extensively engineered to incorporate diverse ncAAs in bacterial expression systems [34, 61]. The pyrrolysyl-tRNA synthetase (PylRS)/tRNA pairs from various archaea have proven particularly versatile, enabling incorporation of hundreds of distinct ncAAs [62, 63]. Notably, PylRS/tRNA pairs exhibit universal orthogonality, functioning in both prokaryotic and eukaryotic hosts. Additional systems include *E. coli*-derived tyrosyl [64–66], leucyl [47, 67–69], and tryptophanyl [43, 44, 70] pairs for eukaryotic expression, a yeast-derived tryptophanyl pair for bacterial hosts [71], and chimeric pairs [72, 73] engineered for orthogonality across multiple expression systems. Using directed evolution, these aaRS/tRNA pairs are engineered to incorporate the desired ncAA with high fidelity and efficiency [31, 33, 34, 37].

The GCE technology has now been successfully implemented in diverse hosts including *E. coli* [41], yeast [64, 74–76], insect [77] and mammalian cell lines [33, 69, 78–80], and also in multicellular organisms including *Caenorhabditis elegans* [81], *Drosophila melanogaster* [82], and rodents [83, 84], providing a broadly applicable platform for precise and programmable protein modification.

While the modularity described above has enabled GCE’s adoption across diverse hosts, its practical implementation remains constrained by several system-level factors intrinsic to the translation machinery. Nonsense (or quadruplet) codon suppression must compete with endogenous release factors or near-cognate tRNAs, imposing an efficiency ceiling that compounds at multiple incorporation sites (Section 4.1). Heterologous expression of orthogonal aaRS/tRNA pairs, particularly at the ncAA loads required for efficient suppression, can impose a measurable metabolic burden and cellular stress on the host, and the ribosome’s own fidelity checkpoints can constrain the orthogonal system [85]. These constraints do not preclude scalability, but they do mean that translating a given chemistry from proof-of-concept to a scalable setting requires system-level, and not merely chemical, optimization — a theme we return to throughout this review (see also Section 6).

It is also possible to target ncAA incorporation at specific sense codons, resulting in stochastic replacement of a particular canonical amino acid residue across the entire proteome (Figure 2C). Such residue-specific, proteome-wide incorporation of a bioorthogonal ncAA serves as the basis for technologies such as bioorthogonal noncanonical amino acid tagging (BONCAT) [86–89] and stochastic orthogonal recoding of translation (SORT) [90, 91], which enable tagging and enrichment of newly synthesized proteins produced in specific cells within a user-defined time window. This can be achieved either by introducing an engineered ncAA-selective variant of an endogenous aaRS into the host cell, which competes with its native counterpart to charge the cognate tRNA (BONCAT), or by using an orthogonal ncAA-selective aaRS/tRNA pair targeted to a sense codon (SORT). It has also been possible to use long-term adaptive laboratory evolution of bacterial cells to stabilize codon–ncAA reassignment at the whole-cell level [92]. However, we will not discuss this topic in further detail, focusing instead on site-specific protein labeling approaches.

## 3 | Chemistries Used for Protein Labeling

Over the last few decades, significant progress has been made in developing bioorthogonal reactions, in which two chemical handles selectively react with each other under physiologically relevant conditions without cross-reacting with functionalities commonly found in biomolecules [9–11, 13, 93–98]. Collectively, these reactions have become a cornerstone of modern chemical biology, enabling countless applications. Many ncAAs containing bioorthogonal handles have been genetically encoded across various domains of life, allowing facile protein labeling selectively at the site of incorporation. The following subsections provide an overview of these chemistries, the available ncAAs harboring these functional groups, and their utility and limitations. It is important to note that none of these reactions are fully inert toward all biomolecules; rather, they exhibit varying degrees of chemoselectivity. They also differ in kinetics, compatibility with living systems, and other parameters that should be carefully considered when selecting a labeling reaction for a specific application.

### 3.1 | Carbonyl-Based Bioconjugation Reactions

Aldehydes and ketones chemoselectively react with alkoxyamines or hydrazides under mildly acidic conditions (pH 4–7), to form stable oxime and hydrazone linkages, respectively (Figure 3A) [99, 100]. Although these reactions are inherently slow, with second-order rate constants on the order of 10^−4^–10^−3^ M^−1^ s^−1^ [101, 102], their kinetics can be significantly accelerated by aniline catalysis [103, 104].

GCE has enabled the site-specific incorporation of many carbonyl-bearing ncAAs (Figure 3C), most notably *p*-acetylphenylalanine (*p*-AcF, **1**) [105]. It was genetically encoded in *E. coli* using the *Mj*TyrRS/tRNA^Tyr^ pair and in eukaryotic cells using *Ec*TyrRS/tRNA^Tyr^ systems [64, 79, 105–107]. The resulting proteins undergo efficient oxime or hydrazone formation with alkoxyamine- or hydrazide-functionalized probes. Additional examples of ketone-containing ncAAs that have been genetically encoded include *m*-acetylphenylalanine (*m*-AcF, **2**; in *E. coli* using *Mj*Tyr pair [106], also the Pyl pair [108]), *β*-diketophenylalanine (dkF, **4**; in *E. coli* using *Mj*Tyr pair) [109], the aliphatic ketone **5** (in *E. coli* using the Pyl pair) [110], and others [47].

An aldehyde-containing amino acid (**3**) has also been incorporated using an engineered Pyl pair [111]. Labeling of **3**-containing proteins with hydroxylamine dyes proceeded efficiently in the presence of aniline catalysis, achieving second-order rate constants of approximately 182 ± 12 M^−1^ s^−1^. In a related study, the same ncAA was applied in reductive amination reactions to generate a stable secondary amine linkage (Figure 3B) [112].

Despite their utility, carbonyl-based conjugations face several practical challenges. They require mildly acidic conditions, particularly when labeling more popular ketone-ncAAs, which are incompatible with many proteins and with cellular environments. In addition, the slow reaction rates often demand high reagent concentrations for efficient labeling. As a result, these chemistries are generally more suitable for in vitro applications on robust proteins such as antibodies.

### 3.2 | Staudinger Ligation

Derived from the classical Staudinger reduction [113], this reaction couples an azide with a triarylphosphine bearing an electrophilic trap—typically an ester—initially forms an iminophosphorane intermediate, which subsequently undergoes intramolecular acyl transfer to yield an amide linkage (Figure 4A) [114]. In the modified “traceless” version of the ligation, a cleavable acyl group attached to the phosphine enables intramolecular acyl transfer to produce a native amide bond while releasing a phosphine oxide byproduct (Figure 4B) [115]. The chemoselective reaction proceeds under mild aqueous conditions, and is protein-compatible. However, the second-order rate constants are relatively modest, typically in the range of 10^−3^–10^−2^ M^−1^ s^−1^ [116].

*p*-azidophenylalanine (*p*-AzF, **6**, Figure 4D) was among the azide-bearing ncAAs to be genetically encoded for site-specific bioorthogonal conjugation using an evolved *Mj*Tyr pair [117]. Its incorporation into recombinant proteins and phage- displayed peptides subsequently enabled labeling through Staudinger ligation with fluorescein-derived phosphines [117, 118]. This residue has also been exploited in Staudinger–phosphite ligation, where symmetric or unsymmetric phosphites react with the azide to yield stable phosphoramidate linkages, enabling site-specific biotinylation and the generation of phosphotyrosine analog (Figure 4C) [119–121]. In a related approach, a Pyl pair was used to incorporate *N*^*ε*^-(*o*-azidobenzyloxycarbonyl)-lysine (*o*-AzbK, **7**, Figure 4D) in *E. coli*, and the resulting proteins were labeled with fluorescent probes via Staudinger ligation [122].

The Staudinger ligation suffers from intrinsically slow kinetics [116], often necessitating high concentrations of phosphine reagents. Other limitations include the susceptibility of phosphines to oxidation and side reactions involving azide reduction. Collectively, these factors have limited its applications mainly to in vitro or cell-surface labeling. Notably, additional azide-containing ncAAs discussed in the subsequent sections are also likely substrates for Staudinger reaction, although its popularity has waned with the development of other facile bioorthogonal reactions targeting azides.

### 3.3 | Cu(I)-Catalyzed Azide–Alkyne Cycloaddition (CuAAC)

The Cu(I)-catalyzed azide–alkyne cycloaddition (CuAAC), or the prototypical “click” reaction, is a highly useful bioorthogonal transformation [98, 123]. This reaction involves the regioselective [3+2] cycloaddition of an organic azide and a terminal alkyne in the presence of a Cu(I) catalyst to form a stable 1,2,3-triazole linkage. The catalytic cycle proceeds through the formation of a copper–acetylide intermediate, which coordinates with the azide to generate a six-membered metallacyclic transition state that collapses to the triazole product (Figure 5A) [124, 125]. CuAAC reactions exhibit excellent bioorthogonality, high conversion, and compatibility with physiological conditions. When assisted by optimized, water-soluble ligands such as BTTES, THPTA, or BTTAA, overall rate constants in the range of 10–200 M^−1^ s^−1^ can be achieved [126].

Many azide-containing ncAAs have been incorporated into proteins through GCE in both prokaryotic and eukaryotic systems, enabling applications of the CuAAC chemistry (Figure 5B). **6** (Figure 4D) and **8** (Figure 5B, exemplify early examples of such aryl-azide-containing ncAAs [64, 65, 79, 107, 108, 117, 122]. Additional examples include **9**, using *Mj*Tyr [127], and Pyl pairs [128]; **9–11** using *Ec*Tyr pairs [129]; **12–17** using various Pyl pairs [130–135]; **12**, **18a-18c**, and **19** using the *Ec*Leu pair [47, 68]; **20** using the *Ec*Trp pair [43]; smaller aliphatic azides using Pyl [136]; 3-azidotyrosine using archaeal [137] or bacterial [138] Tyr pairs, etc. Notably, the picolyl-azide ncAA (**17**) exhibits strong copper-chelating capability, allowing efficient CuAAC under low-copper conditions when combined with water-soluble ligands such as THPTA, thereby enabling live-cell labeling with reduced cytotoxicity [135]. It is important to note that azide-ncAAs – particularly aromatic azides – can be susceptible to metabolic reduction, leading to partial loss of the installed conjugation site [139].

In parallel, many alkyne-functionalized ncAAs have also been genetically encoded and used for CuAAC-based protein labeling (Figure 5C). The phenylalanine derivatives (**21**) and *p*-propargyloxyphenylalanine (*p*-PrF, **22**) were among the earliest to be incorporated in *E. coli* using the *Mj*Tyr pair [127, 140, 141]. **22** was also incorporated in eukaryotic cells using an *Ec*Tyr pair [65, 79, 107]. Additional alkyne-substituted ncAAs include **23**–**24** using polyspecific *Ec*Tyr pair [129]; **25** using the *Ec*Trp pair [43]; **26**–**29** using different Pyl systems [46, 131, 142–145]; **30a**–**30c** using a polyspecific *Ec*Leu pair [47]; smaller aliphatic alkynes using Pyl [136]; etc. Finally, alkyne-containing ncAAs that also contain a photo-crosslinking [146–148], or an electrophilic covalent crosslinking group [149] have been developed. Such dual-functionalized ncAAs enable covalent capturing of interaction partners using either photoactivated or proximity-enabled reactions, followed by the enrichment of the resulting complex via CuAAC-mediated biotinylation.

Despite its broad utility, the requirement for copper catalysis poses intrinsic limitations due to associated cytotoxicity and oxidative protein damage [150, 151]. It also precludes the use of this approach for intracellular applications. These challenges have been mitigated to some extent through the development of water-soluble ligands and copper-chelating azides, which enable efficient conjugation under low-copper conditions [126]. Nevertheless, residual concerns regarding metal-induced stress and incomplete biocompatibility have motivated the development of copper-free click reactions, discussed in the following section.

### 3.4 | Strain Promoted Azide-Alkyne Cycloaddition (SPAAC)

Strain-promoted azide–alkyne cycloaddition (SPAAC) is a copper-free alternative to CuAAC that uses ring strain of cyclooctynes to achieve spontaneous cycloaddition with azides without a metal catalyst (Figure 6A). The reaction proceeds through a concerted [3+2] pathway [152–154], generating stable triazoles with second-order rate constants of ~0.1–1 M^−1^ s^−1^ in aqueous media [20, 155]. These kinetics, combined with excellent biocompatibility, allow selective protein modification in live cells and in vivo settings [156, 157]. SPAAC has become a preferred ligation for site-specific labeling, imaging, and therapeutic conjugation, broadening the chemical space accessible for biological engineering.

Various azide-containing ncAAs (Section 3.2,3.3) have been genetically incorporated to enable SPAAC with strained alkynes [108, 117, 158, 159]. Complementarily, strained alkyne residues such as cyclooctyne-containing lysine (**31**, **32**) [160] and tyrosine derivatives [161] have also been genetically encoded using engineered Pyl pairs (Figure 6B). More strained cyclooctyne variants, including Biocyclo[6.1.0]nonyn-lysine (BCNK, **33**, Figure 6B) [68, 156, 162, 163] and **31** [68, 163–165] have also been encoded using Pyl and *Ec*Leu pairs. These undergo efficient SPAAC with azide probes under physiological conditions for diverse applications.

SPAAC has established itself as one of the most popular methods for site-specific protein labeling in vitro and in cellular contexts. However, moderate reaction rates, low but significant cross-reaction of strained cyclooctynes toward thiol and other functionalities [166], and significant hydrophobicity of cyclooctyne groups are key limitations that restrict its application in complex settings. Continued development of more reactive, yet stable, and accessible cyclooctyne scaffolds is expected to expand the scope of SPAAC labeling.

### 3.5 | Photoclick Cycloadditions

Alkene–tetrazole photoclick cycloadditions rely on UV-induced extrusion of N_2_ from tetrazoles to generate a reactive nitrile imine dipole, which undergoes rapid [3+2] cycloaddition with alkenes to form highly stable pyrazoline adducts (Figure 7A) [167, 168]. The light-triggered nature of this reaction grants temporal and spatial control over labeling events. The reaction is also ‘fluorogenic’, since the tetrazole precursor is weakly emissive while the cycloaddition product exhibits strong fluorescence. Photoclick reactions proceed under mild aqueous conditions with rate constants around 1–100 M^−1^ s^−1^ [169, 170].

Genetically encoded tetrazole- and alkene-bearing amino acids have enabled direct photoclick labeling on proteins across bacterial, yeast, and mammalian systems (Figure 7B). *O*-Allyltyrosine (**34**) was site-specifically incorporated in *E. coli* using an evolved *Mj*Tyr pair, enabling UV-triggered photoclick coupling with diaryltetrazoles for selective protein modification [169–171]. *p*-(2-Tetrazole)phenylalanine (**35**) was also similarly incorporated and used for fluorogenic photocycloaddition with fumarate dyes [172]. Several alkene-bearing lysine derivatives (**36**–**41**) have since been genetically encoded using Pyl pairs [173–181]. More reactive strained alkenes such as (**36**) [177, 182] and spirohexene-lysine (**37**) [176] have also been incorporated using the Pyl platform, with **37** supporting in-cell labeling with excellent rates (~10^4^ M^−1^ s^−1^) using water-soluble tetrazoles [176, 183]. The electron-deficient *N*_*ε*_-acryloyllysine (AcrK, **38**) was efficiently installed in bacteria and eukaryotic cells, which enabled rapid nitrile-imine cycloaddition and fluorescence turn-on within minutes [180, 181]. Photoinduced tetrazole–BCN ligation enabled ultrafast labeling of BCNK (**33**)-encoded proteins in live cells, achieving rate constants of ~3.9×10^4^ M^−1^ s^−1^ (Figure 7C) [179]. Hydrazone sulfones, functioning as stable tautomers of nitrile imines, like-wise supported rapid bioorthogonal ligation on BCNK-tagged nanobodies [178].

Photoclick chemistry has emerged as a versatile platform for rapid, fluorogenic protein labeling. Despite these advances, reliance on UV activation, which is associated with limited penetration and potential phototoxicity constrain its applications. The instability and off-target reactivity of the nitrile imine intermediate is also a concern. Continued development of red-shifted tetrazoles and reactivity tuning will further expand its scope.

### 3.6 | Metal-Catalyzed Protein Coupling Reactions

Transition-metal catalysis has expanded protein bioconjugation using ncAA-encoded handles beyond CuAAC. Palladium- and ruthenium-mediated cross-coupling and olefin metathesis tolerate aqueous conditions, proceed with high chemoselectivity, and unlock bond types inaccessible to native residues (Figure 8).

Early demonstrations used *p*-iodophenylalanine (**42**) incorporated using an evolved *Mj*Tyr pair in *E. coli* [184], for site-specific Mizoroki–Heck coupling [185] with and Sonogashira cross-coupling [186] with alkene and alkyne reagents, respectively, to generate biotin-tagged proteins (Figure 8A,B). Similarly, *meta*-iodophenylalanine (**43**) incorporated using the Pyl system was shown to undergo Sonogashira coupling [187], while *ortho*-iodo- (**44**) and other halogenated phenylalanine analogues remain viable scaffolds for Pd-mediated cross-coupling [187–189]. Recently, *p*-bromopropargyloxyphenylalanine (**45**) was encoded using a polyspecific *Mj*Tyr pair, allowing sequential CuAAC and Sonogashira coupling to build multivalent protein conjugates [190]. Additionally, lysine derivatives bearing alkyne (**26c**, Figure 5C) or iodophenyl (**46**) groups have been incorporated using the Pyl pair and subjected to Pd(NO_3_)_2_-mediated, ligand- and copper-free Sonogashira coupling for site-specific protein labeling [191]. Using the water-soluble ligand 2-amino-4,6-dihydroxypyrimidine (ADHP), **26b** (Figure 5C), containing proteins underwent copper-free Sonogashira arylation in vitro, inside *E. coli*, and on live mammalian cell surfaces [192]. The same iodophenyl handle **42** also enabled Suzuki–Miyaura coupling with boronic-acid probes [188, 193, 194], while *p*-borono-phenylalanine (**47**) introduced via a *Mj*Tyr pair allowed complementary Suzuki labeling using iodinated BODIPY (Figure 8C) [195]. Recently, fluorosulfatetyrosine (**48**) was encoded using a Pyl pair in *E. coli* and labeled through Suzuki coupling with boronic-acid derivatives [196]. Also, S-allylhomocysteine (**49**) was incorporated and used for Ru-catalyzed cross-metathesis for chemoselective protein modification, establishing a genetically encoded handle for cross-metathesis on folded proteins (Figure 8D) [197, 198]. In addition, *p*-PrF (**22**) has been labeled using a CuI/TMEDA-mediated aqueous Glaser–Hay oxidative coupling with alkyne-functionalized fluorophores to form rigid diyne-linked protein conjugates under mild conditions (Figure 8E) [199].

Overall, metal-mediated coupling reactions vastly expand the repertoire of site-specific protein bioconjugation approaches using ncAAs. However, limitations such as the reliance on transition-metal catalysts and their potential toxicity, modest yields, need for elevated temperatures, slower kinetics, and limited biocompatibility continue to restrict their broader applications.

### 3.7 | Inverse-Electron-Demand Diels-Alder Cycloadditions (IEDDA)

Inverse-electron-demand Diels–Alder (IEDDA) cycloaddition between electron-poor tetrazines and strained alkenes/alkynes has emerged as a versatile bioorthogonal reaction for protein conjugation [200]. The reaction proceeds through a concerted [4+2] cycloaddition with immediate N_2_ release, forming a 4,5-dihydropyridazine intermediate that converts to the stable 1,4-dihydropyridazine product in protic media, with rate constants commonly in the 10^3^–10^4^ M^−1^ s^−1^ range (Figure 9A) [201]. Such exceptional kinetics arise from the high ring strain of dienophiles and the strongly electron-deficient tetrazine core, enabling efficient labeling at low reagent levels under physiological conditions. Free of metal catalysts and compatible with living systems, IEDDA offers excellent speed, selectivity, and fluorogenic potential, making it the preferred chemistry for live-cell imaging [202–206], multi-site labeling, protein tracking, and therapeutic conjugation [207, 208].

Both tetrazine- and strained alkene–bearing ncAAs have been genetically encoded, enabling ultrafast catalyst-free protein labeling (Figure 9C). As early examples, tetrazine-bearing Phe analogues (**50**, **51a**) were incorporated using the *Mj*Tyr systems in *E. coli*, supporting fast site-specific in vivo IEDDA ligation with cyclopropane-fused trans-cyclooctene (sTCO)-modified probes [209–211]. Tetrazine amino acids (**52a**–**52d**) were later encoded in eukaryotic systems using Pyl pairs, enabling faster and more robust IEDDA labeling [212]. Subsequently, *β*-tetrazine variants (**53a**–**53c**) were encoded by evolving the Pyl pair, enabling rapid and quantitative site-specific labeling in cells [213]. Most recently, improved tetrazine-bearing ncAA **54** was encoded using the Pyl pair that shows superior labeling kinetics (k_2_ >10^7^ M^−1^ s^−1^) [214].

Complementarily, strained alkene-containing lysine derivatives (**36**, **37**, **39**; **55**, **56**, Figures 7B and 9C) [176, 177, 182, 215–217], *trans*-cyclooctene lysines (**57**–**60**) [68, 206], and the strained alkyne BCNK (**33**) [68, 163, 206] were encoded via engineered Pyl pairs, providing rapid tetrazine ligation in vitro and in live mammalian cells [217]. Unlike IEDDA that relies on strained alkene/alkyne dienophiles, isocyanide-bearing ncAAs (**61**, **62**) were genetically encoded using engineered chimeric pairs, enabling catalyst-free [4+1] tetrazine cycloaddition for site-specific labeling (Figure 9B) [218].

IEDDA has emerged as one of the most powerful platforms for protein bioconjugation, enabling rapid labeling kinetics, fluorogenic reaction, and excellent chemoselectivity. The chemistry is still constrained by tetrazine susceptibility to reduction [219] and TCO isomerization [220], particularly under in vivo conditions, which can constrain its utility under specific circumstances. Future advances in stabilizing the reagents, without compromising the reaction rates, will further expand the scope of IEDDA, especially for in vivo applications.

### 3.8 | 1,2-Aminothiol-Based Reactions

1,2-Aminothiol functionalities represent a versatile bioorthogonal handle capable of undergoing several distinct ligation reactions under mild conditions. One of the earliest demonstrations involved their reaction with thioesters, enabling native chemical ligation (NCL)–type coupling through a chemoselective trans-thioesterification followed by intramolecular S-to-N acyl transfer (Figure 10A) [221]. In one of the early examples, aminothiol-bearing amino acids (**63**, **64**, Figure 10E) were genetically encoded in *E. coli* using the Pyl pair, allowing conjugation of ubiquitin through NCL [221]. In a related strategy, a thioester-activated aspartic acid (**64a**) was genetically encoded via an engineered Pyl pair, enabling site-specific NCL with 1,2-Aminothiol-derived probes for protein labeling [222]. A complementary reactivity was later reported with aldehydes, where the same 1,2-aminothiol handle condensed to form stable thiazolidine linkages under physiological conditions (Figure 10B) [223].

1,2-aminothiols also selectively and efficiently react with nitriles, a reaction referred to as the nitrile–aminothiol (NAT) click reaction. The transformation proceeds via nucleophilic attack of the thiol on the electrophilic carbon of the nitrile, forming a thioimidate intermediate that subsequently undergoes intramolecular attack by the neighboring amine to yield a stable thiazoline adduct (Figure 10C) [224]. This mechanism operates efficiently under physiological conditions. Building on this chemistry, **63**–**65** were incorporated into proteins using evolved Pyl pairs, and subsequently labeled with nitrile-functionalized fluorophores [224]. More recently, Huber and coworkers coupled the biosynthesis of activated nitrile-bearing ncAAs (**66**–**68**, Figure 10E) with GCE and incorporated using an optimized Pyl variants, enabling NAT-click conjugation and live-cell labeling under physiological conditions [225]. A related approach replaced the nitrile partner with a 2-((alkylthio)(aryl)methylene)malononitrile (TAMM) electrophile, which reacts with 1,2-aminothiols through a thiol–vinyl sulfide exchange followed by cyclization and elimination of dicyanomethanide to form a stable dihydrothiazole adduct (Figure 10D) [226]. In this strategy, the genetically encoded **65** was employed, which upon methoxyamine-mediated deprotection, released the free 1,2-aminothiol and enabled site-specific labeling with TAMM-derived fluorophores [226].

Overall, the NAT-click and TAMM-based ligations offer rapid and selective conjugation with second-order rate constants in the range of 1–9 M^−1^ s^−1^, enabling efficient site-specific modification [224, 226, 227]. Despite these advantages, their application remains largely confined to in vitro and cell-surface labeling, due to side reactions with endogenous metabolites such as pyruvate, and glutathione/cysteine.

### 3.9 | Azo-Coupling Reaction

Azo coupling is a classic electrophilic aromatic substitution reaction between electron-rich aromatic groups and aryldiazonium ions, which has been used for protein conjugation at tyrosine residues [228]. However, less electrophilic aryldiazoniums do not react with tyrosine at physiological pH, but can couple with more electron-rich aromatic groups with enhanced nucleophilicity, forming the basis of chemoselective azo-coupling reactions. This reaction offers tunable kinetics and selectivity, forms a stable linkage, and proceeds under physiological conditions.

A 2-naphthol–derived amino acid (**69**, Figure 11C) was incorporated in *E. coli* using an evolved *Mj*Tyr pair, which underwent selective azo coupling with electron-rich diazonium reagents under neutral pH (Figure 11A) [229]. With excess reagent, the reaction proceeds with pseudo–first-order kinetics, displaying a rate constant of (8.4 ± 0.4) × 10^−3^ s^−1^ and affords stable azo linkages. A more nucleophilic ncAA, 5-hydroxytryptophan (5HTP, **70**, Figure 11C) was encoded via an engineered *Ec*Trp pair in ATMW1 *E. coli* and eukaryotic cells, enabling the chemoselective rapid azo-coupling reaction (CRACR) (Figure 11B) [230]. 5HTP under-went rapid, site-specific conjugation with different diazonium reagents exhibiting second-order rate constants between 2 and 6×10^4^ M^−1^ s^−1^, underscoring its selectivity and tunability. It has since been used to generate diverse protein conjugates, including for full-length antibodies [42, 44, 230–232]. The resulting azo linkages can also be selectively cleaved by mild reductants such as dithionite, offering a rare example of an “unclickable” bioconjugation strategy.

Despite their efficiency and operational simplicity, azo-coupling and CRACR reactions remain largely confined to in vitro applications, as diazonium reagents can be unstable and occasionally cross-react with reactive native residues [233].

### 3.10 | Oxidative Coupling Methods

Oxidative coupling methods exploit the chemoselective redox activation of electron-rich aromatic residues such as phenylenediamines, phenols, and 5-hydroxyindoles to generate reactive electrophilic intermediates that couple efficiently with nucleophiles such as anilines under mild conditions.

Several aromatic residues that serve as redox-active coupling handles have been genetically encoded for oxidative bioconjugation. A virus capsid displaying *p*-aminophenylalanine (**71**), incorporated using an engineered *Mj*Tyr pair, was selectively labeled with synthetic peptides containing a phenylenediamine residue through NaIO_4_-mediated oxidation (Figure 12A) [234]. Alternatively, 5HTP (**70**) can be activated using a milder oxidant, potassium ferricyanide, which underwent efficient aniline coupling to afford site-specific protein modification (Figure 12B) [235]. Recently, electrochemical oxidation (eCLIC) has been applied for site-specific labeling of 5HTP residues. This method enables controlled C─N or C─C bond formation using primary or tertiary aniline coupling partners, respectively (Figure 12C) [236]. Similarly, a photoredox-catalyzed bioconjugation (PhotoCLIC) was implemented on site-specifically encoded 5HTP residues, using a combination of methylene blue and blue/red LEDs to trigger selective coupling of aromatic amine reagents (Figure 12D) [237]. In addition to aniline and 5HTP, the electron-rich catechol moiety of 3,4-dihydroxyphenylalanine (DOPA, **72**) has also been genetically encoded using a chimeric PylRS–PheRS (chPheRS) system, and subsequently subjected to ferricyanide-mediated oxidative coupling with anilines (Figure 12E) [72, 238]. The encoded DOPA (**72**) residue can also undergo strain-promoted oxidation-controlled cyclooctyne-1,2-quinone (SPOCQ) cycloaddition with BCN derivatives, following ferricyanide-mediated oxidation (Figure 12F) [238]. In a complementary strategy, BCNK (**33**) was genetically incorporated and reacted with 1,2-quinone formed via NaIO_4_-mediated oxidation of catechol derivatives, establishing mutually reactive oxidative conjugation partners for site-selective protein labeling [239].

Despite their versatility, oxidative bioconjugation methods face practical limitations in cellular settings. The oxidative conditions, though mild, may not be compatible with sensitive proteins.

### 3.11 | Conjugate-Addition

Conjugate addition of reactive nucleophiles such as thiols onto electrophilic *α*,*β*-unsaturated carbonyl residues can generate stable covalent conjugates under physiological conditions. GCE can be used to site-specifically introduce either the electrophile or the reactive nucleophile, enabling the use of this chemical approach for precise protein labeling.

An early example of this approach used the site-specific incorporation of phenylselenocysteine (**73**) using an engineered *Mj*Tyr pair in *E. coli* [240, 241]. Mild oxidation of **73** with H_2_O_2_ efficiently generates a dehydroalanine (Dha) electrophile, which undergoes rapid and selective Michael addition with thiol nucleophiles to form defined thioether conjugates (Figure 13A). In a parallel approach, the same Dha electrophile was accessed by incorporating *O*-phosphoserine (**74**) using the orthogonal SepRS9/tRNA^Sep^ system with the evolved elongation factor (EF)-Sep21, followed by the *β*-elimination to generate Dha (Figure 13B) under alkaline conditions [242]. In contrast to preceding strategies that generate the Michael acceptor in situ, direct incorporation of acrylolysine (AcrK, **38**, Figure 7B) installs an intrinsic acrylamide on the protein, enabling efficient site-specific PEGylation through conjugate addition of PEG thiols under aqueous conditions (Figure 13C) [180].

In a complementary strategy, the reactive nucleophile can also be introduced on the protein by GCE. For example, incorporating a protected homocysteine (**75**) using an engineered mutated Pyl pair (Figure 13D) introduced a latent thiol [243], which can be uncaged enzymatically using penicillin G acylase (PGA). The resulting thiol underwent efficient Michael addition with maleimide-functionalized fluorophores to furnish site-specific conjugates. In a related strategy, a photocaged cysteine (**76**) was incorporated site-specifically using an engineered *Ec*Leu pair. Light-mediated decaging unveiled a free cysteine that participated in conjugate addition generating site-specific homogeneous antibody–drug conjugates (Figure 13E) [244, 245]. These approaches are useful for proteins lacking free thiols, such as antibodies, and where introducing engineered cysteine residues is problematic due to their oxidative capping during expression/secretion and/or the scrambling of native disulfide bonds.

Similarly, a photocaged selenocysteine (**77**) was incorporated via an engineered *Ec*Leu system, enabling selective protein labeling after photo-decaging via conjugate addition with electrophilic probes (Figure 13E) [246, 247]. Exploiting the higher nucleophilicity of the liberated selenol, photocaged selenocysteine platform also enabled rapid and selective alkylation with electrophilic tags, achieving chemoselective protein modification even in the presence of solvent-exposed cysteine residues [248]. Direct selenocysteine incorporation has also been achieved in engineered *E. coli* [249]. Finally, ncAAs containing a secondary amine side chain have also been incorporated using a Pyl pair, followed by their conjugate addition onto a quinone electrophile, generated by tyrosinase oxidation of *p*-cresol [250].

Although conjugate-addition chemistry provides rapid site-specific modification of certain proteins in vitro, it is typically not suitable for complex biological settings. In addition, these approaches are often restricted by the need for harsh chemical activation steps, and incompatibility with free cysteine thiols on the target protein.

### 3.12 | Emerging Site-Specific Ligation Strategies

The need for protein bioconjugation is remarkably diverse. The demands vary depending on the protein to be modified, the biological context, the degree of chemoselectivity required, whether single or multiple labels are needed, and whether the goal is to generate small amounts of conjugates as probes or to produce biotherapeutics at scale economically. Although many site-specific bioconjugation strategies have been developed, no single method is ideal for all applications, each having its own pros and cons. However, having multiple options with complementary strengths provides a versatile toolbox capable of meeting this wide range of needs. These diverse demands have continued to drive the development of emerging techniques, which are summarized in this section.

A cyclopropenone-lysine derivative (**78**) was recently incorporated into proteins using a Pyl pair [251]. The encoded cyclopropenone reacted efficiently with phosphines bearing a pendent biotin-substituted amine motif to form a ketene ylide intermediate, which was then intramolecularly trapped by the amine to yield a covalently ligated product (Figure 14A). In a complementary approach, asparaginyl endopeptidase (AEP)–mediated ligation was combined with GCE to achieve site-specific protein conjugation. A dipeptidic GG motif was introduced site-specifically by encoding a lysine derivative bearing azidoglycine – glycine on the *ε*-amine (**79**), using an evolved Pyl pair (Figure 14B) [252]. Staudinger reduction unveiled the GG nucleophile, which was then recognized by an engineered AEP (*Oa*AEP1) to ligate NGL-tagged probes or proteins through a transient thioester intermediate, yielding defined conjugates.

A dithiolane-containing phenylalanine (**80**) was genetically encoded using a polyspecific *Mj*Tyr pair and selectively conjugated with organoarsenic probes, enabling rapid and reversible protein labeling in vitro and in live cells (Figure 14C) [253]. The same dithiolane ncAA was also applied to protein immobilization on gold nanoparticles, yielding homogeneous protein–metal conjugates without additional chemical modification [254].

## 4 | Site-Specific Multi-Labeling of Proteins

Site-specific protein labeling using GCE has traditionally been limited to a single modification per polypeptide, due to the low efficiency of co-incorporating more than one ncAA. However, the ability to introduce the same label at multiple predefined sites, or distinct labels at different sites within a protein, is highly desirable for next-generation applications. Recent advances in GCE technology now make this possible.

### 4.1 | Attaching the Same Label at Multiple Sites

Competition with translation termination mediated by release factors compromises the efficiency of ncAA incorporation at nonsense codons, which had traditionally precluded multisite incorporation. However, the development of genomically recoded *E. coli* strains lacking release factor 1 (RF1) [255, 256] eliminates this competition at the UAG codon, enabling ncAA incorporation at multiple sites [257, 258]. Further optimization of orthogonal aaRS/tRNA pairs [258], or the use of endogenous aaRS/tRNA pairs for ncAA incorporation [259], has additionally improved multisite incorporation efficiency, facilitating down-stream bioorthogonal attachment of multiple labels to a protein [259, 260]. Using this strategy, up to 10 *p*-AzF (**6**) residues were introduced into ELP-GFP and subsequently labeled with a fatty acid via CuAAC. Increasing lipidation enhanced albumin binding and slowed clearance in an animal model, providing a modular chemical approach for tuning the in vivo half-life of protein therapeutics [260]. In mammalian cells, development of improved expression systems [107, 261–266], as well as the overexpression of an engineered release factor that promotes readthrough [182] has facilitated multisite incorporation of the same ncAA.

### 4.2 | Attaching Multiple Distinct Labels

Site-specific attachment of multiple distinct labels to the same protein requires incorporating different ncAAs with distinct conjugation handles, using mutually orthogonal aaRS/tRNA pairs that decode different nonsense or quadruplet codons (Figure 15A) [267]. For this strategy to succeed, cross-reactivity must be avoided at multiple levels: the orthogonal aaRS/tRNA pairs must not cross-react with each other, the ncAA substrates must be selective for their respective aaRS, and the labeling chemistries must be mutually compatible (Figure 15A–C). One of the earliest demonstrations of this strategy used incorporation of *p*-AzF (**6**) and propargyl-lysine (ProK, **26a**) in *E. coli* using *Mj*Tyr and Pyl pairs, at UAG and UAA codons, respectively (Figure 15D) [45]. The resulting protein carried an azide and an alkyne at defined positions, which could be individually labeled using CuAAC, although simultaneous labeling of both handles was not shown. A contemporaneous study similarly incorporated *p*-AzF and ProK, using *Mj*Tyr (AGGA) and Pyl (UAG) pairs, respectively, using an evolved orthogonal ribosome to enhance decoding of quadruplet codons [49]. The site-specifically introduced azide and alkyne handles were subsequently linked together using CuAAC to introduce an intramolecular crosslink (Figure 15B). The *Mj*Tyr (UAG) and Pyl (UAA) pair were also used in *E. coli* to introduce *p*-AcF (**1**) and AzK (**12**), respectively, enabling subsequent dual labeling via oxime condensation and SPAAC [268]. In another study, *p*-PrF (**22**) and CpK (**55**) were incorporated using *Mj*Tyr (UAG) and Pyl (AGUA) systems, followed by dual labeling using CuAAC and IEDDA, respectively [269]. The same strategy was later used to incorporate a tetrazine ncAA (**50**) and a norbornene-ncAA (**56**), which do not react with each other, but can be sequentially labeled using a BCN and a hyperreactive dipyridyl-tetrazine reagent (both IEDDA), respectively [51]. The *Mj*Tyr (UAG) + Pyl (UAA) system has also been used for co-incorporating *p*-AzF and tetrazine-ncAA (**52a**) that enable dual labeling via SPAAC and IEDDA [270]. The oxidative eCLIC [236] and PhotoCLIC [237] conjugations on 5HTP (**70**) are compatible with SPAAC and IEDDA chemistries, which have been used together for dual labeling proteins following simultaneous incorporation of 5HTP and ncAAs such as AzK (**12**) or CpK (**55**) into proteins.

In mammalian cells, *Ec*Tyr (UAG) and Pyl (UAA) pairs were first used to install *p*-AcF (**1**) and AzK (**12**, Figure 5B), respectively [261]. The encoded carbonyl and azide groups facilitated oxime condensation and SPAAC, generating antibody–drug conjugates bearing both a therapeutic payload and an imaging probe (Figure 15C). The same strategy was also later used to install CpK (**55**) and *p*-AzF (**6**) at UAG and UGA sites, respectively, enabling orthogonal IEDDA and SPAAC reactions [46]. The Pyl (UGA) and *Ec*Leu (UAG) pairs were also used together to co-incorporate CpK (**55**) and the azide-ncAA **18c**, followed by dual labeling [47]. Two mutually orthogonal Pyl pairs were recently used to site-specifically incorporate ProK (**26a**) and TCO*K (**58**), allowing dual labeling of proteins, including the SynNotch receptor, via CuAAC and IEDDA chemistries, respectively [271]. In yeast, *Ec*Tyr (UAG) and *Ec*Leu (UGA) pairs have been used to co-incorporate *p*-AzF and ProK for dual labeling [272].

Advancing beyond dual labeling, a triple-suppression system combining *Mj*Tyr (UAG), Pyl (UAA), and *Ec*Trp (UGA) pairs enabled simultaneous incorporation of *p*-AzF (**6**), CpK (**55**), and 5HTP (**70**) into a single protein in *E. coli*, allowing mutually selective SPAAC, IEDDA, and CRACR labeling for site-specific triple labeling (Figure 15F) [42]. A similar approach was extended to mammalian cells, where *Ec*Trp (UGA), *Ec*Tyr (UAG), and Pyl (UAA) were combined to incorporate three different bioorthogonal ncAAs simultaneously for multiple labeling, including the synthesis of a full-length antibody site-specifically conjugated to two different drugs [44]. Using a novel *Mj*Tyr pair that decodes UAU as the initiator codon, and combining with two mutually orthogonal Pyl pairs that decode UAG and UAA, it has also been possible to simultaneously incorporate three distinct bioorthogonal ncAAs *p*-AcF, ProK, and *m*-AzF (**8**) [273]. A recent strategy developed the novel ncAA *m*-TAF (**81**, Figure 15E), bearing both a tetrazine and an azide group, allowing labeling with two distinct entities on the same side chain. This ncAA was further co-incorporated with *p*-AcF (**1**) to site-specific triple functionalization [274].

Although site-specific multi-labeling of proteins through ncAA incorporation has emerged as a powerful technology, current systems still suffer from declining suppression efficiency as more nonsense codons are introduced. Additional challenges include a limited set of bioorthogonal ncAAs that meet all the criteria, including chemical stability in biological setting and commercial availability, robust incorporation through multiple distinct aaRS/tRNA pairs, and mutually compatible attachment chemistries that are efficient and work well in complex biological settings. These challenges become particularly acute as we venture beyond dual incorporation and labeling.

## 5 | Applications of Site-Specific Protein Bioconjugation

Over the past two decades, site-specific protein labeling using ncAA mutagenesis has been widely adopted for diverse applications [31, 32, 34, 36, 275–277]. Given the breadth of these applications and their extensive coverage in prior reviews, we focus here on two of the most popular aspects, probe attachment for biological studies, and therapeutic applications.

### 5.1 | Attaching Probes for Biological Studies

Site-specific bioconjugation using ncAAs has become a powerful tool for mechanistic studies that demand precise control over the placement of a biophysical probe, which cannot be often addressed using traditional approaches. Additional advantages include the option to target numerous internal sites, the relatively small size of the ncAAs that minimizes perturbation to the target protein, and the ability to perform labeling in complex settings (Figure 16A). Countless publications have reported fluorescence-labeling of a target protein in vitro or on the cell-surface, modified with one bioorthogonal ncAA. Examples include precise labeling of membrane receptors such as GPCRs [278, 279], ion channels [280], and receptor tyrosine kinases [281], as well as complexes such as ribosomes [282], and virus particles [157, 283–289], enabling imaging and tracking applications. The majority of these applications employed SPAAC and IEDDA chemistries, and to a lesser extent CuAAC and carbonyl-conjugation. Several factors contributed their popularity, including robust incorporation and commercial availability of the corresponding ncAAs (e.g., **1**, **6**, **12**, **26a**, **33**, **55**, **58**, **52d**, etc.), as well as the selectivity and efficiency of the conjugation reactions, particularly for SPAAC and IEDDA.

However, for labeling applications inside live cells, where the demand for selectivity and efficiency is much higher, the IEDDA reaction has been consistently superior. Intracellular fluorescent probe labeling of proteins has been achieved using ncAAs containing BCN (**33**), TCO (e.g., **58**), and tetrazine (e.g., **52d**) handles [156, 164, 204, 212, 290, 291], enabling imaging applications. The impressive kinetics of the IEDDA reaction, as well as its fluorogenic nature have both contributed to its success. This approach has also been used to attach spin labels on proteins for EPR measurements [213], and to introduce an inhibitor near the active site of an enzyme through a photo-switchable linker for achieving reversible light regulation of its activity in live cells [292].

Förster resonance energy transfer (FRET) is a powerful method for monitoring protein conformational changes, complex assembly, and other dynamic processes, requiring strategic placement of donor and acceptor fluorophores (Figure 16B) [293, 294]. The flexibility of site selection offered by ncAA technology has made it particularly valuable for such dual-probe attachment. Early examples of this approach combined ncAA mutagenesis with other labeling methods, such as cysteine modification [295], tetracysteine tags [296], or self-labeling protein tags [297], to introduce the FRET pair. For single-molecule FRET measurements, the same ncAA can be used at two distinct sites, followed by stochastic labeling of the FRET pair, since the individual molecules labeled with both the donor and the acceptor can be tracked and measured [282, 298–300]. However, it is also possible to attach two distinct fluorophores using two different ncAAs [51, 268, 301, 302]. Combining dual ncAA labeling with self-labeling protein tags, it has also been possible to introduce three different fluorophores on a GPCR, enabling sophisticated 3-color single-molecule FRET experiments [303]. Precise labeling of the influenza HA2 subunit and subsequent FRET studies revealed reversible transitions among three conformational states during membrane fusion [304], while analogous labeling of NUP98 permitted distance-sensitive FRET measurements that mapped segment distributions and dynamics inside the nuclear pore [305].

Together, these strategies establish site-specific labeling as a powerful toolkit for resolving protein motions and interactions with molecular precision, setting the stage for increasingly quantitative and mechanistically grounded imaging studies.

### 5.2 | Next-Generation Biotherapeutics

Antibody–drug conjugates (ADCs) combine antibody targeting specificity with a potent cytotoxic payload. Their clinical success depends on loading drugs at precisely defined positions with controlled stoichiometry, but conventional conjugation to native lysines or cysteines generates heterogeneous mixtures with variable drug-to-antibody ratios, leading to inconsistent pharmacokinetics and efficacy [306, 307]. GCE overcomes this by installing a chemically unique handle at a predetermined antibody position, enabling homogeneous drug attachment through bioorthogonal chemistries (Figure 16C). Importantly, the small size of ncAA residues allows installation at many more internal sites than competing site-specific approaches, providing unmatched flexibility. Representative examples include oxime-based conjugation on *p*-AcF (**1**) [308, 309]; SPAAC on *p*-AzF (**6**) [310] and AzK (**12**) [311]; IEDDA on CpK (**55**) [208]; [4+2] cycloaddition on cyclopentadiene-derived lysines (**82**, **83**) with maleimide–drug payloads [312, 313]; electrochemical oxidation of 5HTP (**70**) for one-step ADC assembly [236]; and combining CRACR on 5HTP with SPAAC on LCA (**19**) for attaching two distinct payloads on a single antibody [44]. Additionally, photocaged cysteine (**76**) incorporation followed by light-mediated decaging has enabled conjugate-addition-based ADC formation, a strategy particularly useful for antibodies where introducing engineered cysteines is problematic [244]. Several ADCs developed using this approach have reached phase 1/2 clinical trials, including ARX517 from Ambrx [314] and STRO-002 from Sutro Biopharma [315].

GCE-enabled site-specific conjugation has also been applied to tune the pharmacokinetic properties of protein drugs through controlled attachment of PEG chains or other polymers. First examples of this strategy used oxime conjugation on *p*-AcF to PEGylate hGH [316] and FGF21 [317]. AzK-based SPAAC PEGylation of murine IL-4 yielded conjugates with increased serum half-life [318], while related studies on IL-2 [319] and IFN*α*2a [320] similarly produced long-acting cytokines with improved tolerability. Beyond pharmacokinetic improvement, site-specific PEGylation can serve a functional purpose. Using GCE, Ptacin et al. generated a library of IL-2 variants with ncAAs at different positions and screened PEG-conjugated forms for altered receptor specificity [321]. One variant, THOR-707, carried a PEG chain positioned to selectively block IL-2R*α* engagement while preserving IL-2R*β*/*γ* signaling, eliminating Treg expansion and enabling selective CD8^+^ T-cell and NK-cell activation with potent anti-tumor activity.

Traditional bispecific antibodies are generated through genetic fusions, which restricts the accessible topologies. Chemical conjugation using ncAAs overcomes this limitation, enabling diverse topologies by choosing the attachment sites and linker chemistry (Figure 16F). Schultz et al. provided early demonstrations, using *p*-AcF (**1**) for oxime-based construction of bispecific antibodies [322] and their conjugates [323]. More recently, tetrazine-bearing ncAA **52d** was used to construct homo- and hetero-oligomers of nanobodies with flexible topology using the fast IEDDA ligation [324]. Importantly, the nanobody oligomers produced in this way exhibited enhanced SARS-CoV-2 neutralization compared to conventionally linked homodimers, demonstrating that the topological diversity accessible through chemical conjugation can directly translate into improved therapeutic efficacy.

Site-specific bioconjugation through GCE has been extensively applied to engineering viral vectors (Figure 16D). For adeno-associated virus (AAV), the most promising vector for human gene therapy in vivo [284, 325], GCE-installed azide handles have been used to site-specifically couple retargeting peptides [326–328], polymers [329, 330], and other ligands [331, 332] onto capsids via SPAAC, yielding vectors with improved targeting and delivery performance and reduced immunogenicity. This approach was further extended to synthesize precise AAV-protein conjugates via the IEDDA chemistry [330, 333], such as highly tissue-specific AAV-nanobody conjugates, and site-specific attachment of two distinct cargos on AAV capsid at different ncAA residues [330]. Beyond AAV, influenza A virus modified with AzK was ligated to antigenic peptides via SPAAC to produce intranasal vaccines eliciting potent CD8^+^ T-cell responses [334], and a pseudotyped lentiviral vector containing an AzK-labeled VSV-G protein was functionalized through SPAAC for retargeting as well as single-virus-particle tracking applications [335].

The efficiency of homology-directed repair (HDR) by CRISPR-Cas9 can be limited in part by low local concentration of the donor DNA template at the cleavage site [336]. Ling et al. addressed this issue by site-specifically conjugating oligonucleotides to Cas9 incorporating the ncAA **9** using SPAAC [337]. The resulting Cas9–oligonucleotide conjugates recruited donor DNA to the target site through base pairing, markedly increasing HDR efficiency in human cells and mouse zygotes, demonstrating that chemical modification of genome-editing proteins through GCE can complement nucleic acid engineering strategies.

GCE-mediated bioconjugation has also been used to expand the chemical diversity of phage-displayed peptide libraries, which are routinely used to identify lead compounds that bind therapeutically relevant targets [288, 338]. By incorporating azide-functionalized ncAAs into phage-displayed peptides and applying CuAAC with diverse alkyne-functionalized small molecules, chemically augmented libraries can be constructed that incorporate nonpeptidic pharmacophores [287], expanding the scope of phage display for therapeutic peptide discovery.

Finally, ncAA-enabled bioconjugation has recently been applied to the emerging field of targeted protein degradation (TPD; Figure 16E) [339, 340]. A major bottleneck in exploiting the ~700 known E3 ubiquitin ligases for PROTAC development is the lack of specific ligands for most of them. Three contemporaneous studies addressed this challenge by using GCE to site-specifically install bioorthogonal ncAAs on E3 ligases (or E2 enzymes), followed by intracellular click conjugation with a tethered neo-substrate binder to create covalent proximity-inducing constructs that can be evaluated for targeted degradation [341–343]. By systematically varying the ncAA placement, these approaches enable mapping of functional degradation interfaces at single-residue resolution on the native E3 surface in live cells, without requiring E3-specific ligands. These studies extended beyond well-characterized E3 ligases such as CRBN and VHL to ligases with no known ligands (e.g., SPOP) [341], and even demonstrated E3-independent degradation through upstream E2 conjugating enzymes [342, 343].

### 5.3 | Biomaterials and Biosensors

GCE has also enabled precise chemical customization of protein-based biomaterials and biosensors, allowing controlled installation of reactive or reporting functionalities at defined sites without perturbing global structure [344–347]. Such site-specificity overcomes the heterogeneity inherent to traditional labeling, supporting predictable assembly, enhanced stability, and programmable function. In the biomaterials space, CuAAC coupling of azide-functionalized gold nanoparticles to *p*-PrF (22) encoded in bacterial pili generated protein nanowires with ~170-fold higher electronic conductivity, demonstrating sequence-defined production of conductive biomaterials with genetically programmable electronic functionality [348]. SPAAC coupling between DNA polymers and *p*-AzF-containing proteins produced hybrid DNA–protein cages with tuneable dimensions [349]. IEDDA-mediated immobilization using tetrazine-bearing ncAAs (**51a**) on sTCO-modified surfaces yielded biomaterial interfaces with defined loading and orientation; notably, the speed of tetrazine–sTCO ligation improved the retention of enzyme structure and activity upon immobilization by limiting surface exploration and encounter with denaturing hotspots [350, 351].

In the biosensing context, BCNK (33)-incorporated amyloid precursor protein was labeled by IEDDA with a tetrazine fluorophore, generating a molecular-beacon biosensor that selectively reports proteolytically released amyloid-*β* peptides in live cells [352]. A complementary biosensing strategy used *p*-AzF to site-specifically label proteins inside living cells with membrane-permeant, environment-sensitive dyes via SPAAC, enabling real-time readout of conformational signaling events [353].

## 6 | Conclusion and Outlook

Site-specific protein bioconjugation through GCE has shifted the field from opportunistic targeting of native residues to highly precise molecular engineering, enabling homogeneous conjugates for imaging, mechanistic studies, therapeutics, biomaterials, and virus engineering. The approach now supports fast, mutually orthogonal chemistries and multi-site installation of distinct bioorthogonal conjugation handles, broadening what can be built and measured with remarkable precision.

Yet key challenges remain. Although single-site ncAA incorporation is generally facile, certain sequence contexts can substantially reduce the efficiency of ncAA incorporation. However, this is less problematic for bioconjugation applications than for other uses of GCE, since there is generally flexibility to reposition the incorporation site. Furthermore, computational tools such as iPASS can guide selection of sites for high suppression efficiency [354]. Multisite incorporation remains a more significant challenge, as suppression efficiency drops with each additional nonsense codon. Improved incorporation systems — including optimized orthogonal aaRS/tRNA pairs in genomically recoded *E. coli* [257–259], enhanced expression platforms in mammalian cells [262–264] — are important to overcoming this bottleneck. Relatedly, the long-term stability of orthogonal aaRS/tRNA pairs in their host strains — an important consideration for long-term culture, in vivo persistence, or manufacturing-scale applications — remains comparatively underexplored; the ongoing transition from plasmid-based expression to genomically integrated orthogonal translation systems, which can enhance stability and performance, is a promising path forward on this front [258, 355–357].

In addition, although many ncAAs have been reported, not all exhibit reliable incorporation efficiency, and the practical chemoselectivity of the installed bioorthogonal handles can vary depending on the target protein, the biological setting, and whether conjugation is performed on purified protein or in complex environments. Among the available chemistries, IEDDA involving TCO, BCN, or tetrazine handles stands out as the most compatible with live-cell and in vivo applications, owing to its exceptional kinetics and biocompatibility; additional conjugation chemistries with comparable in vivo performance would be welcome additions to the toolkit. Off-target incorporation of ncAAs at endogenous nonsense codons, although occurring at low levels, can generate significant proteome-wide background that complicates sensitive applications. Emerging strategies to address this include the development of more selective decoding systems such as RNA codon expansion using programmable pseudouridine (Ψ) codons [358], as well as spatially restricted incorporation using designer membraneless organelles that confine suppression to specific mRNAs [305]. Finally, although the field has achieved remarkable chemical diversity, the vast majority of ncAAs are incorporated using the *Mj*Tyr and Pyl platforms; populating the ncAA toolbox available through additional orthogonal aaRS/tRNA systems would expand both the accessible chemical scope and the capacity for multisite incorporation using mutually orthogonal pairs.

Despite these challenges, the continued integration of GCE with novel bioorthogonal reactions is expected to deliver increasingly programmable, multifunctional protein architectures for diverse biological, biomedical, and biomaterial applications.

## Figures and Tables

**FIGURE 1 |:**
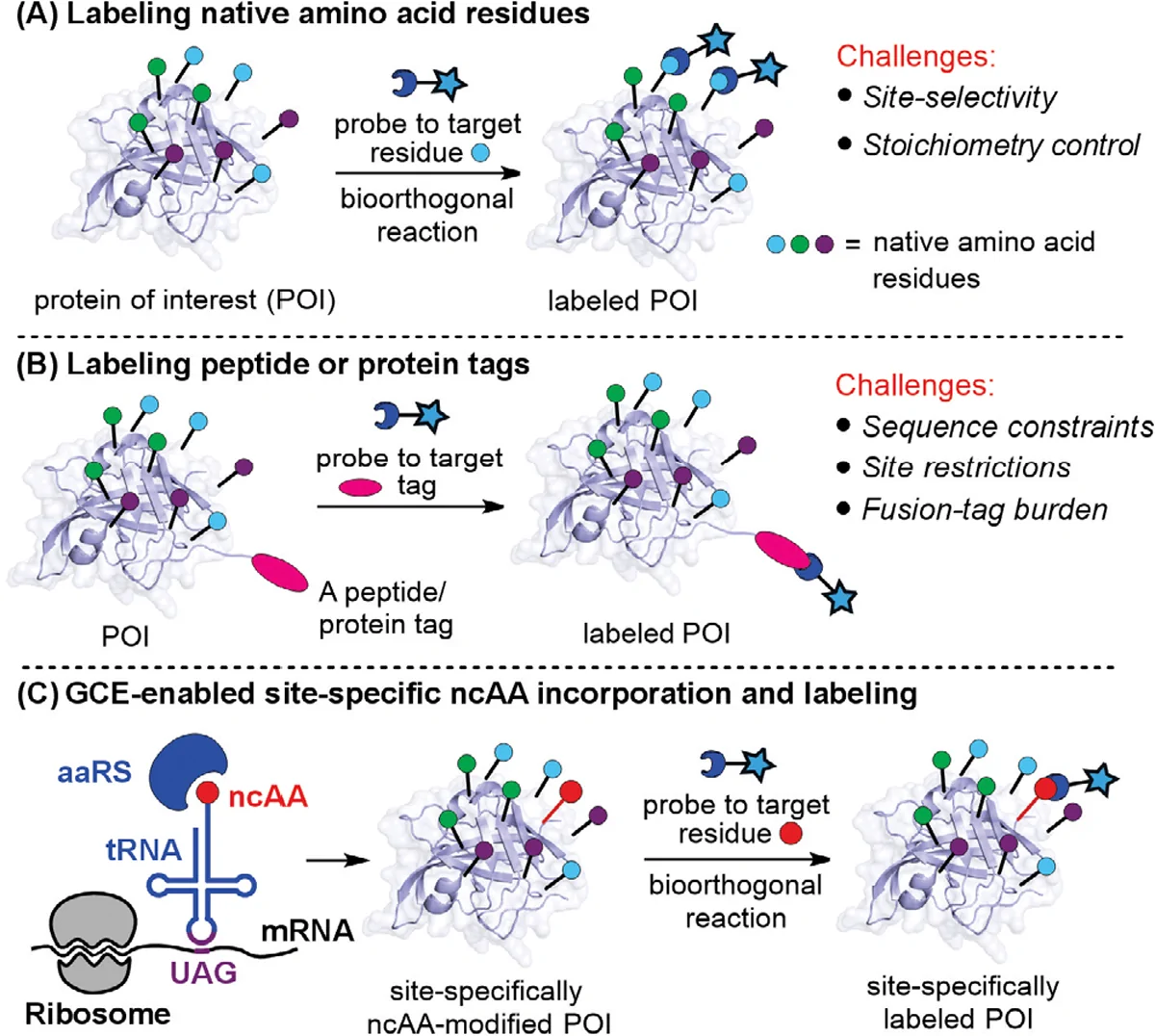
Strategies for site-specific protein labeling.

**FIGURE 2 |:**
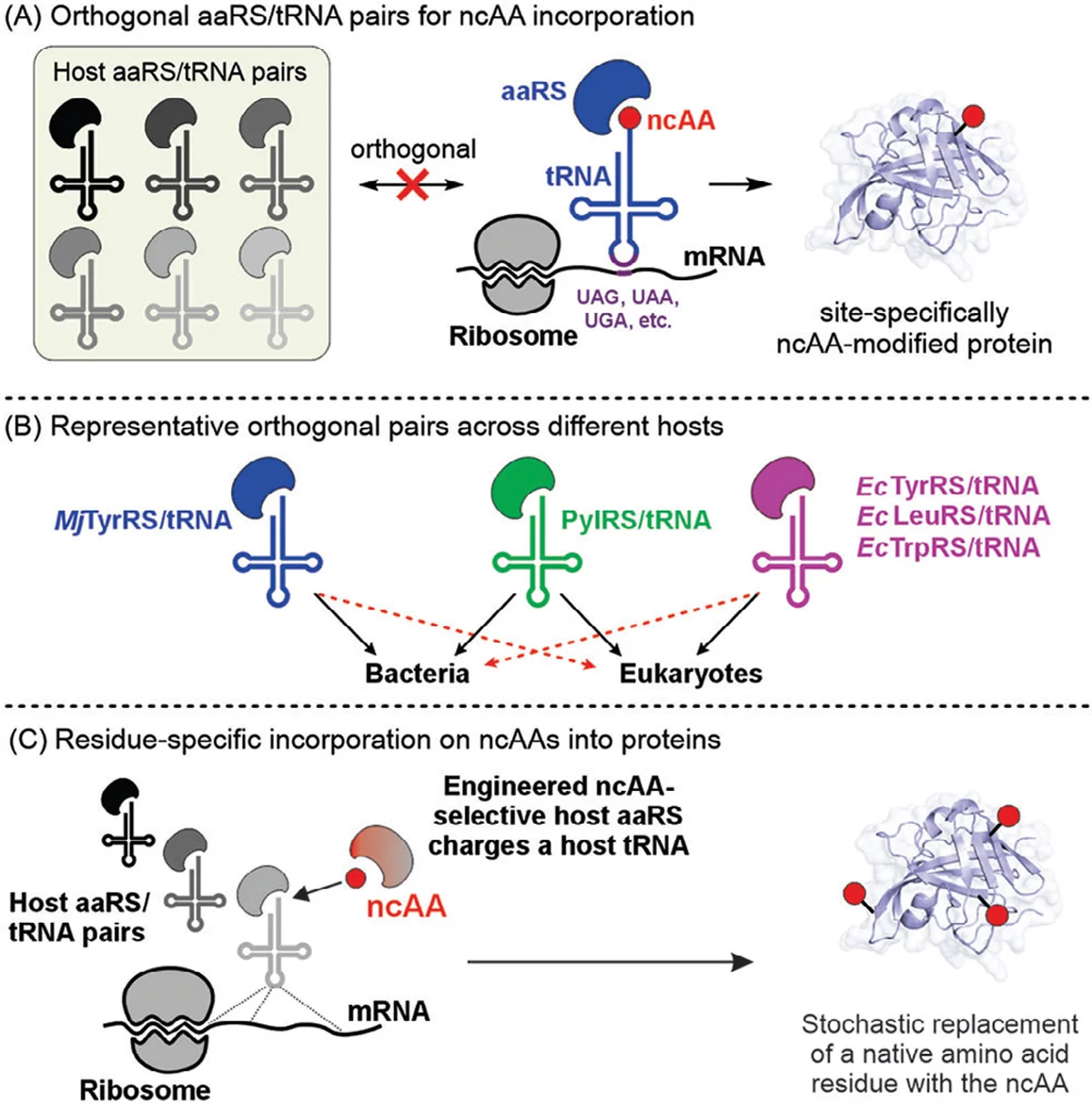
Expanding the genetic code for the site-specific ncAA incorporation.

**FIGURE 3 |:**
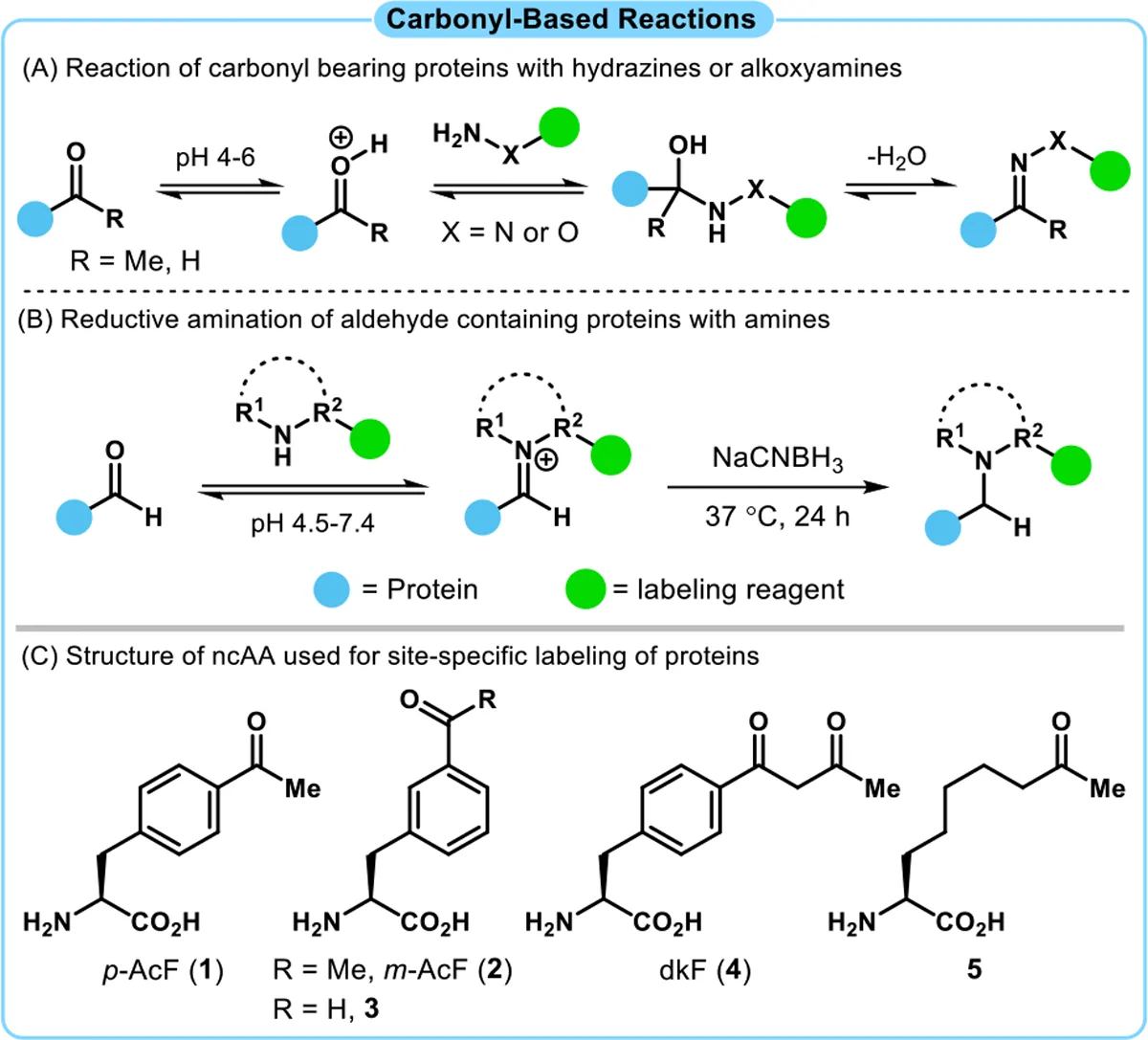
Carbonyl-mediated protein labeling.

**FIGURE 4 |:**
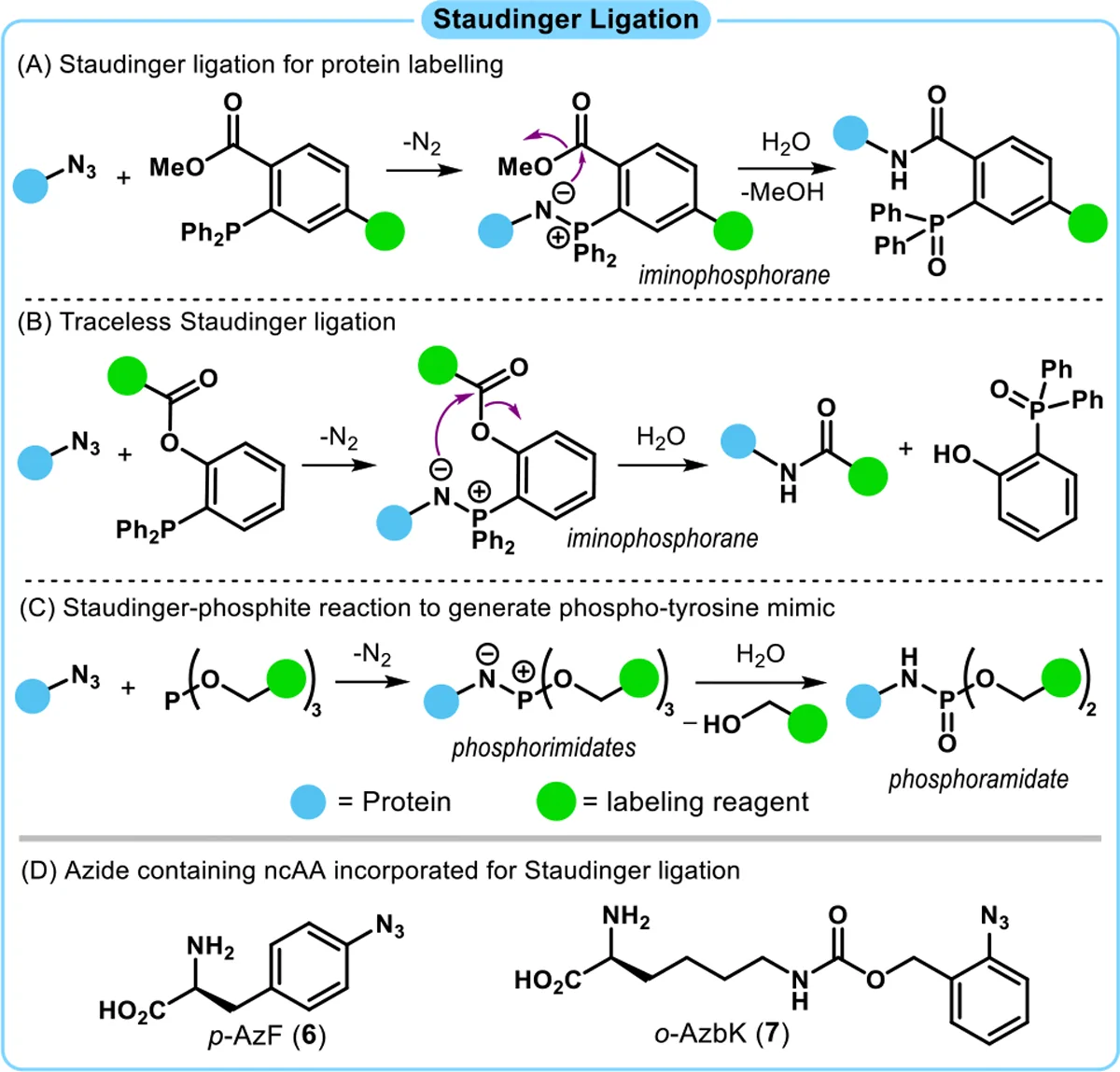
Staudinger-ligation-based labeling strategies.

**FIGURE 5 |:**
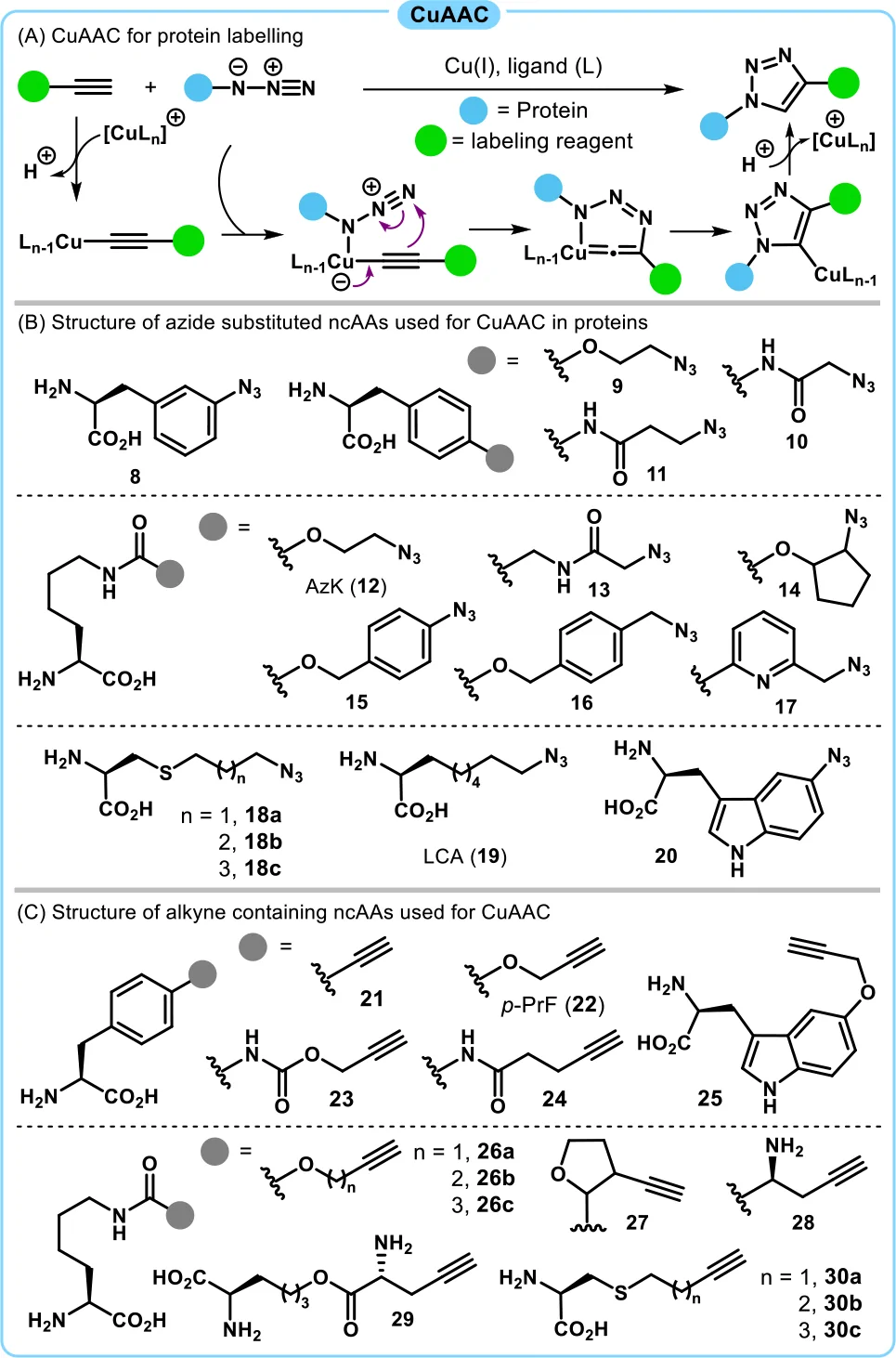
CuAAC-based labeling strategies.

**FIGURE 6 |:**
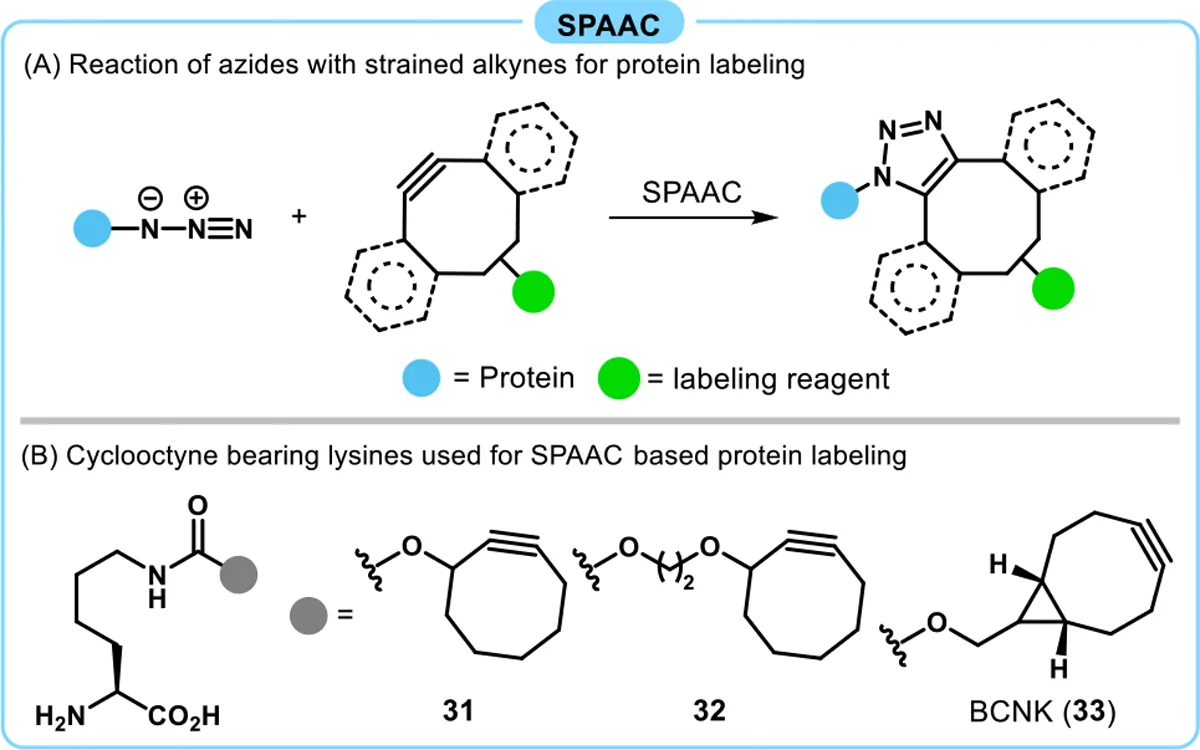
SPAAC-mediated site-specific protein labeling.

**FIGURE 7 |:**
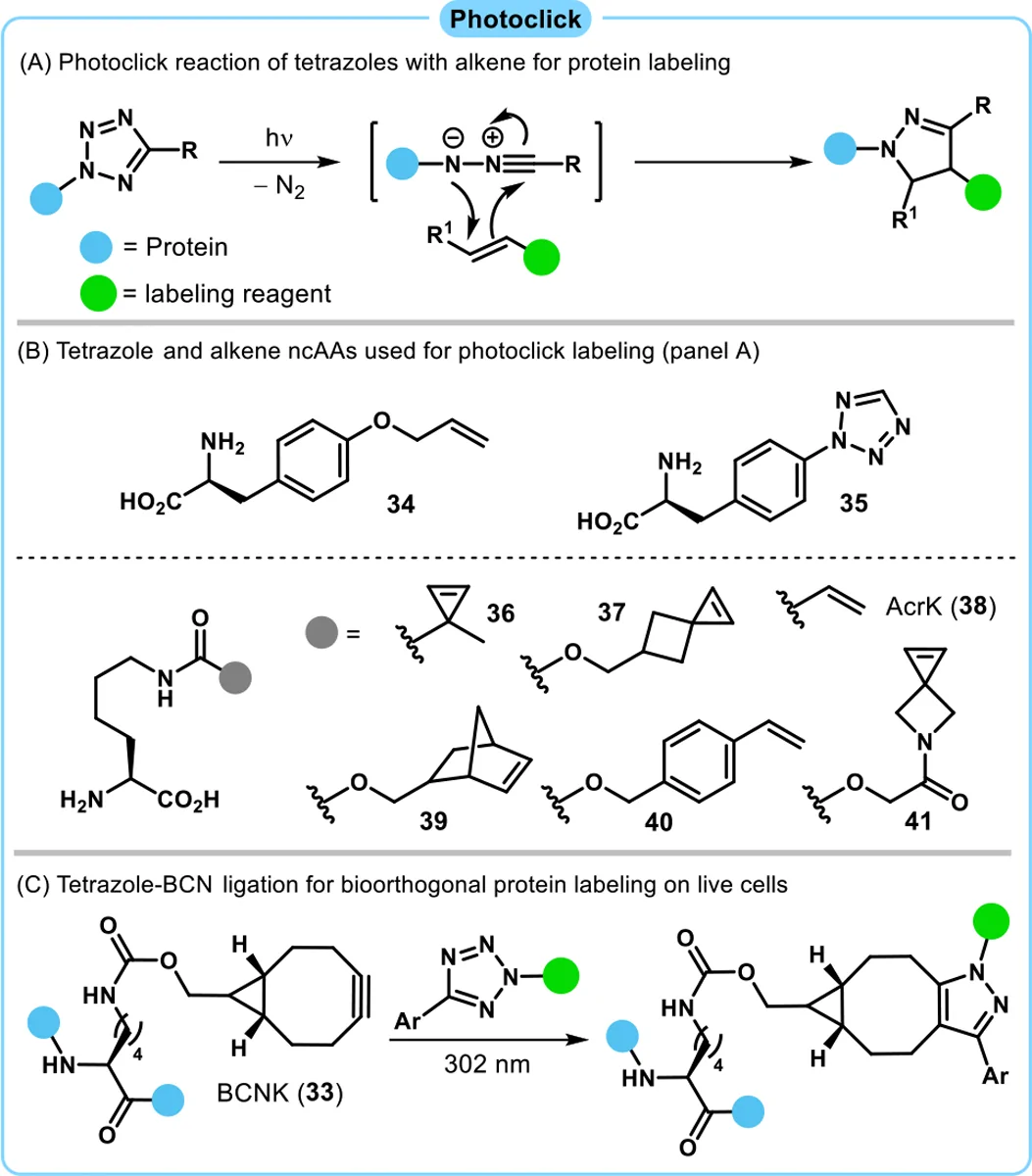
Photoclick mediated labeling strategies.

**FIGURE 8 |:**
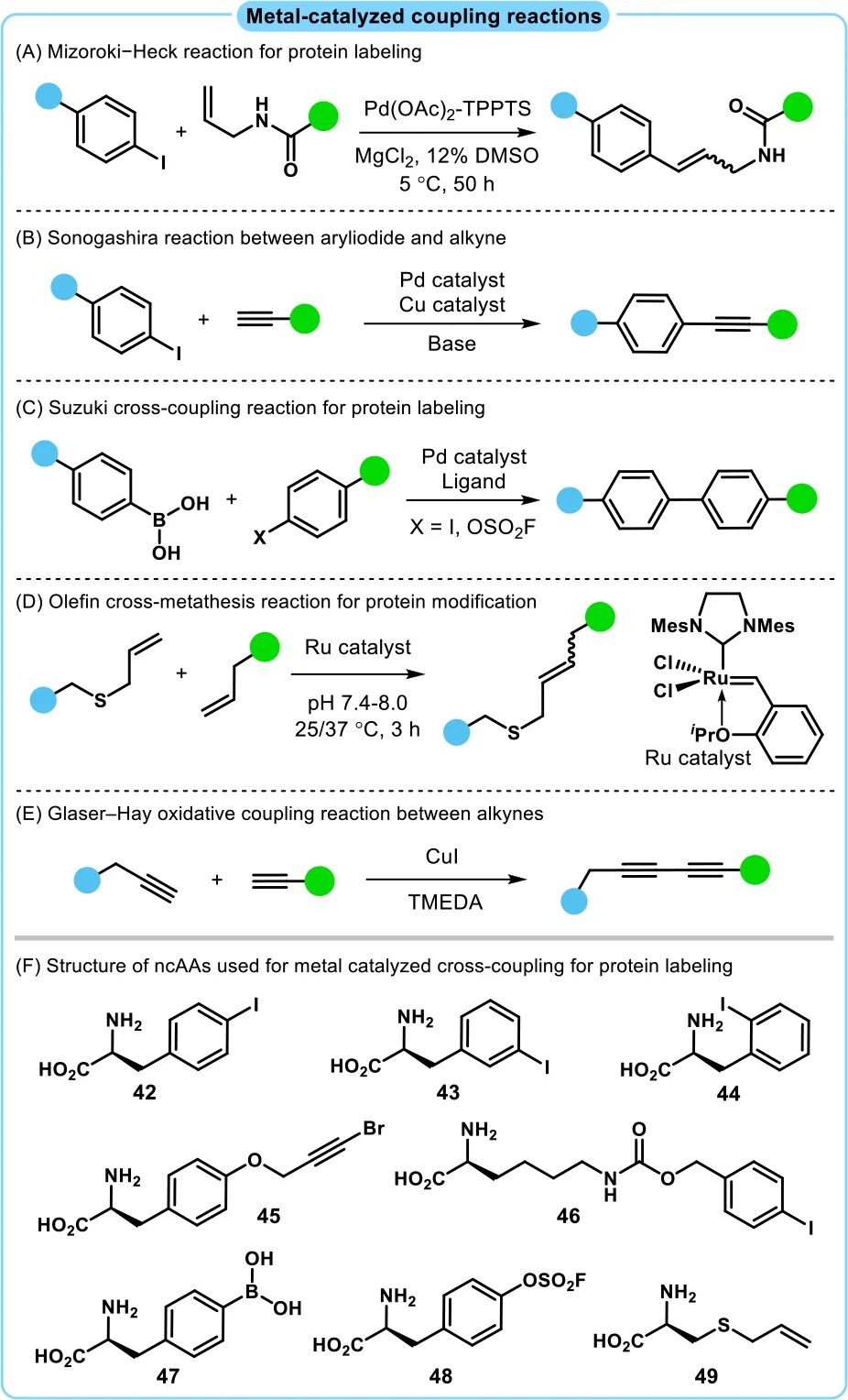
Metal-mediated cross-coupling and metathesis.

**FIGURE 9 |:**
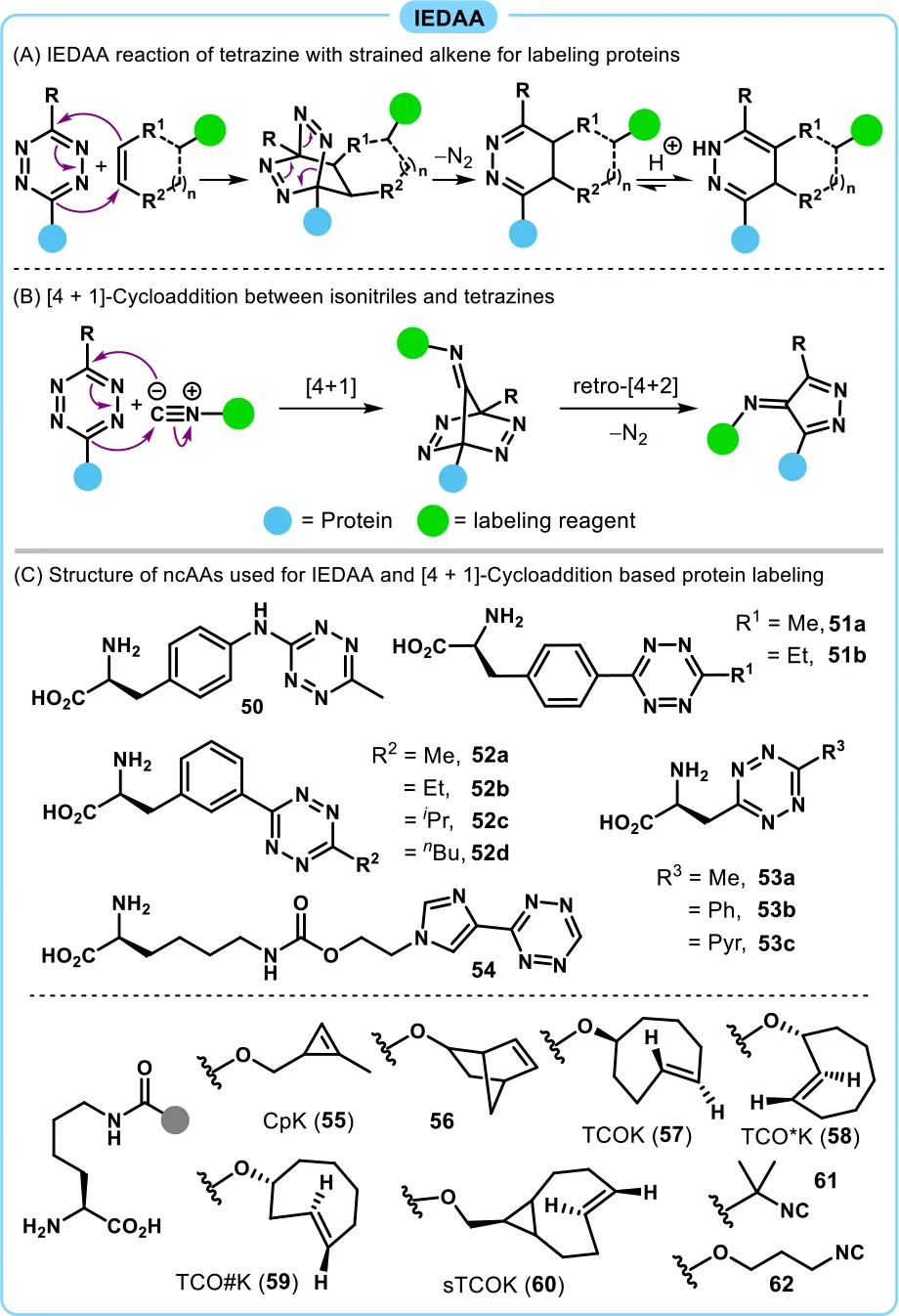
Tetrazine based ligation strategies.

**FIGURE 10 |:**
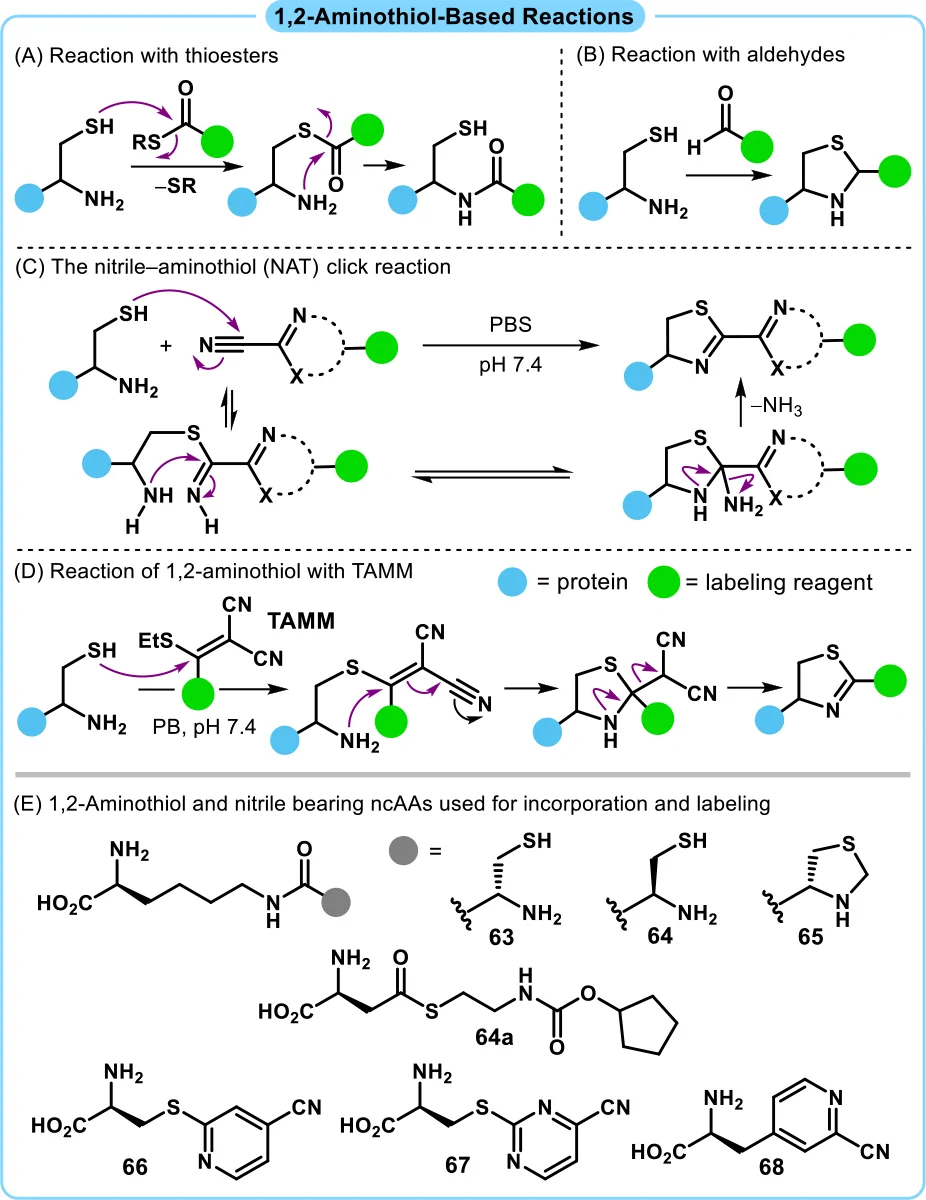
1,2-Aminothiol condensation reactions on proteins.

**FIGURE 11 |:**
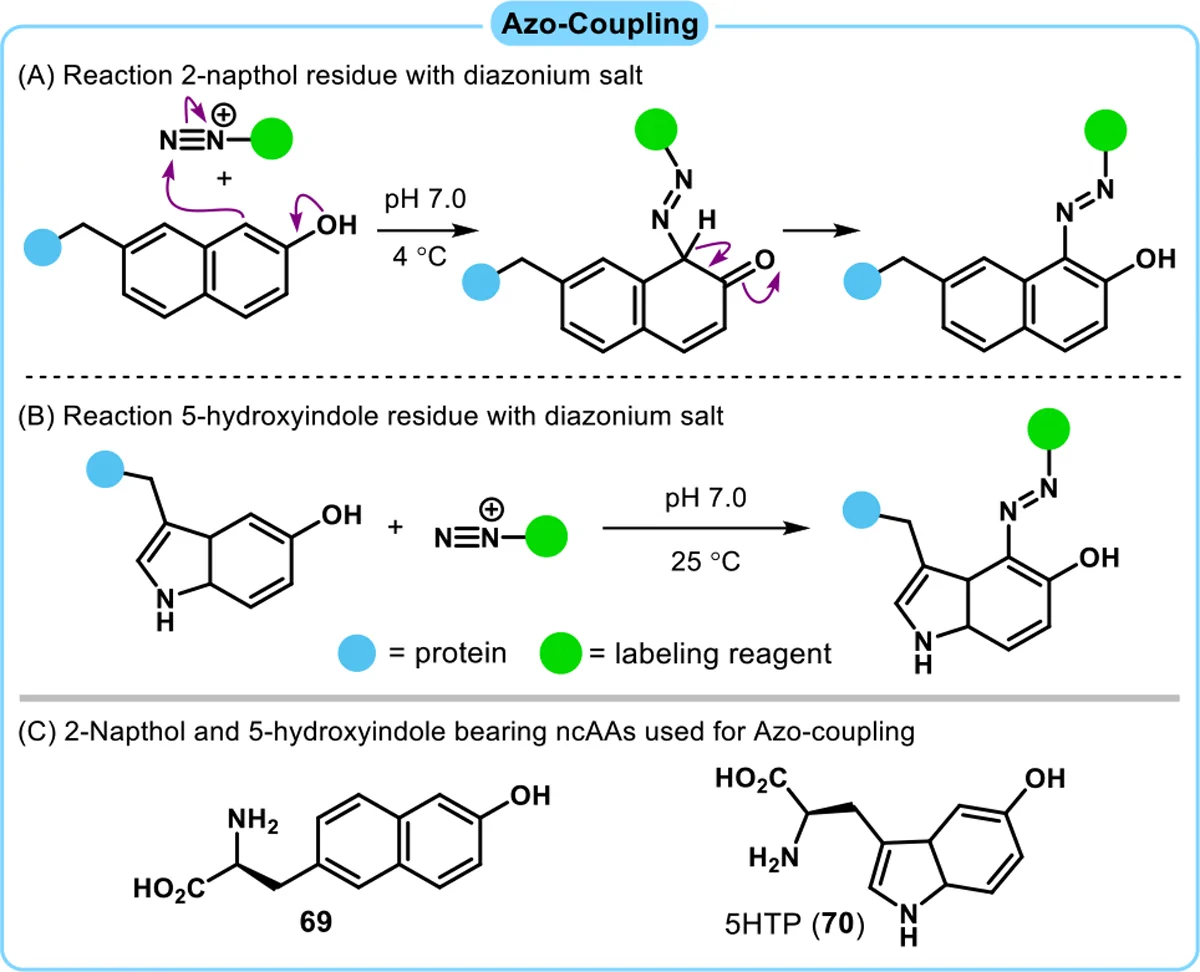
Azo-coupling reactions on proteins.

**FIGURE 12 |:**
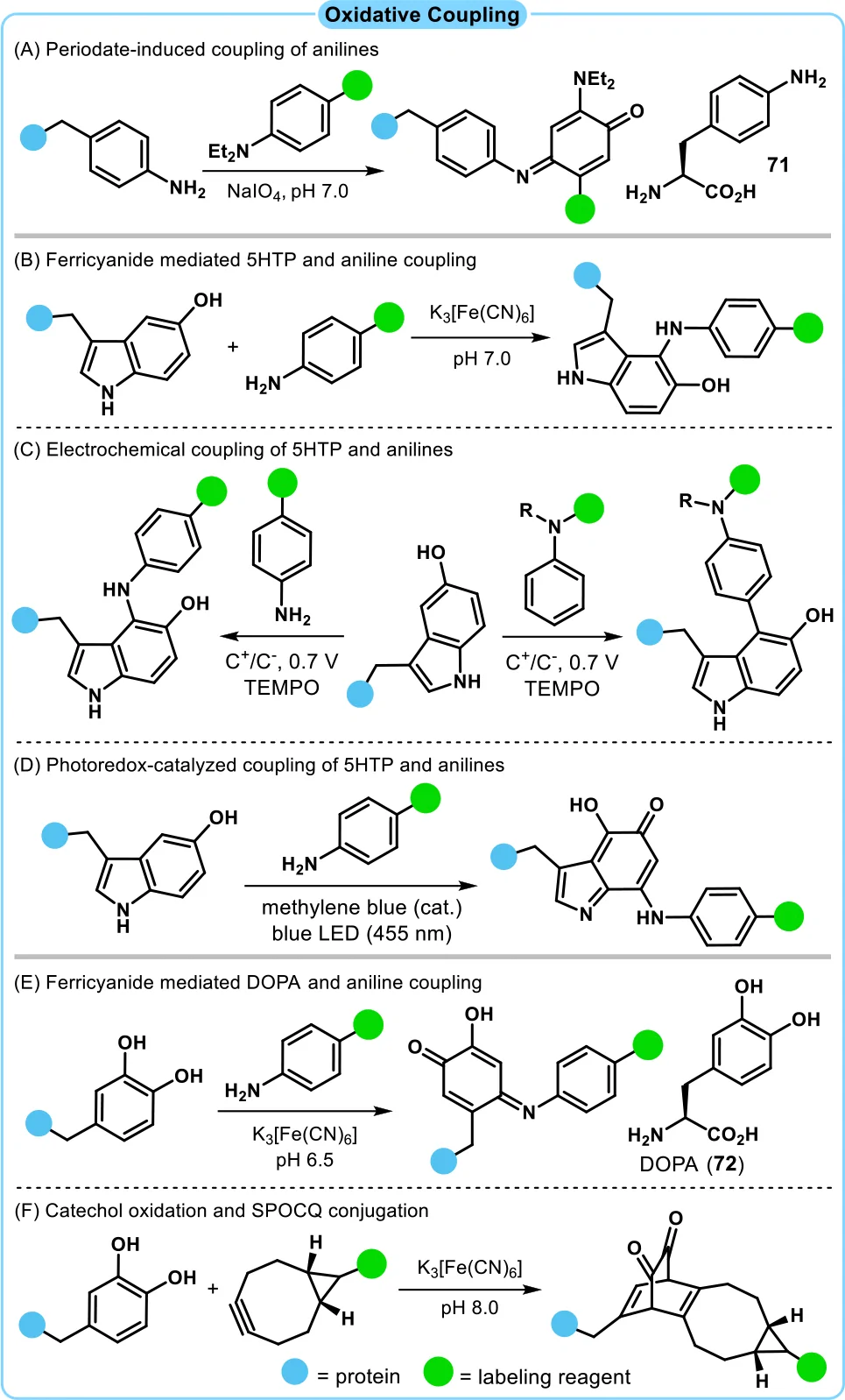
Redox-driven protein bioconjugation.

**FIGURE 13 |:**
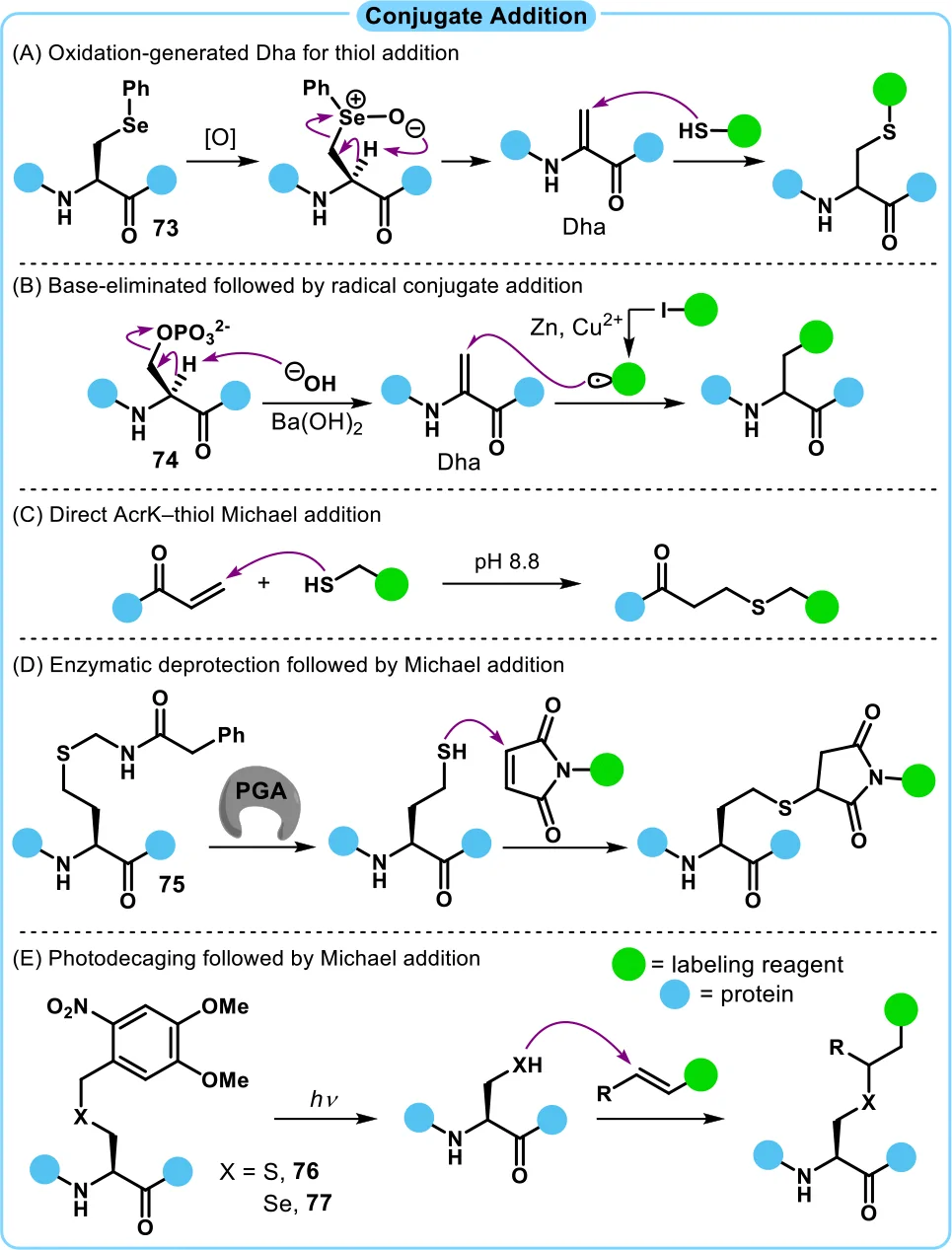
Conjugate-addition routes for protein labeling.

**FIGURE 14 |:**
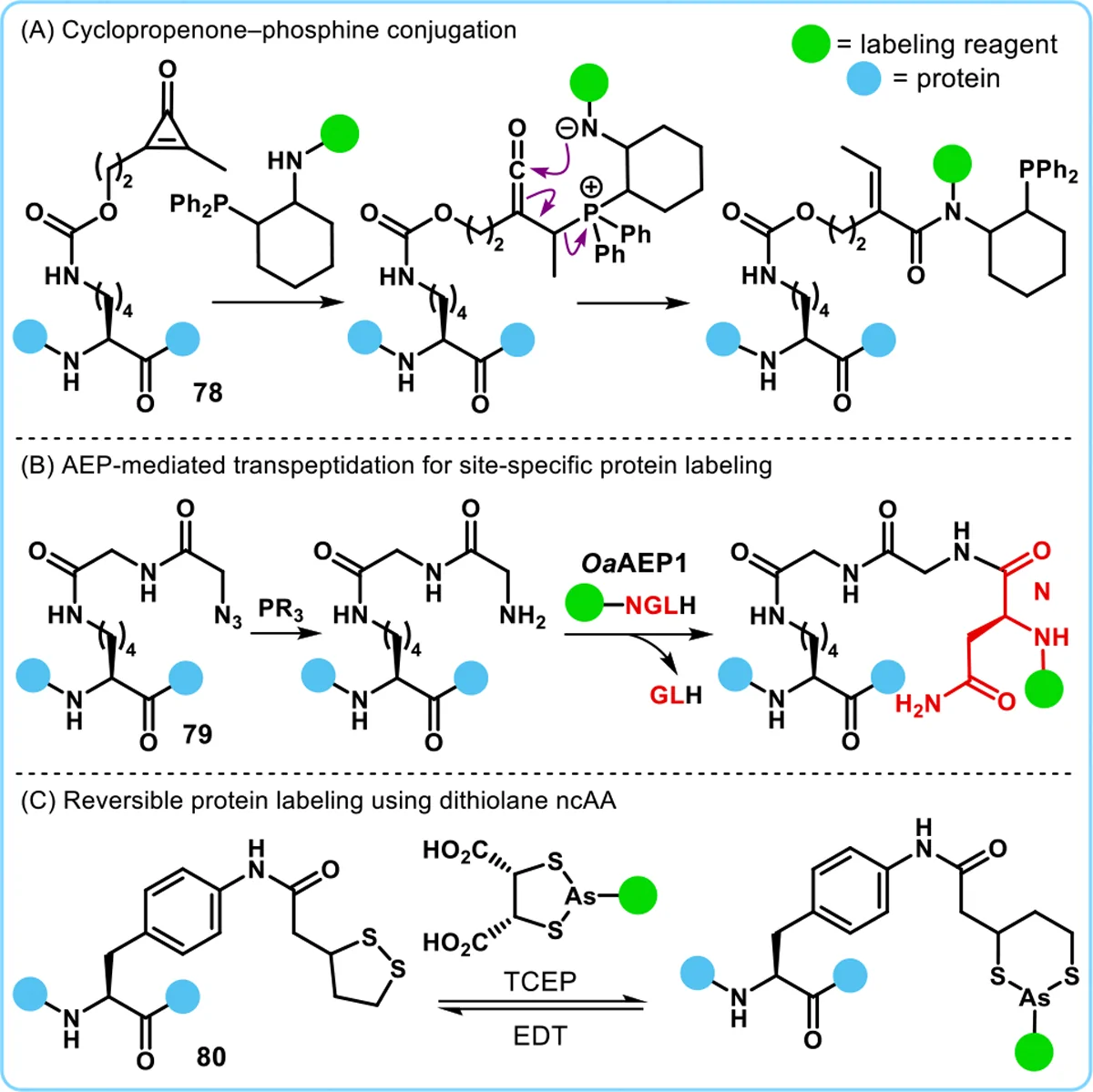
Emerging ligation strategies for site-specific protein conjugation.

**FIGURE 15 |:**
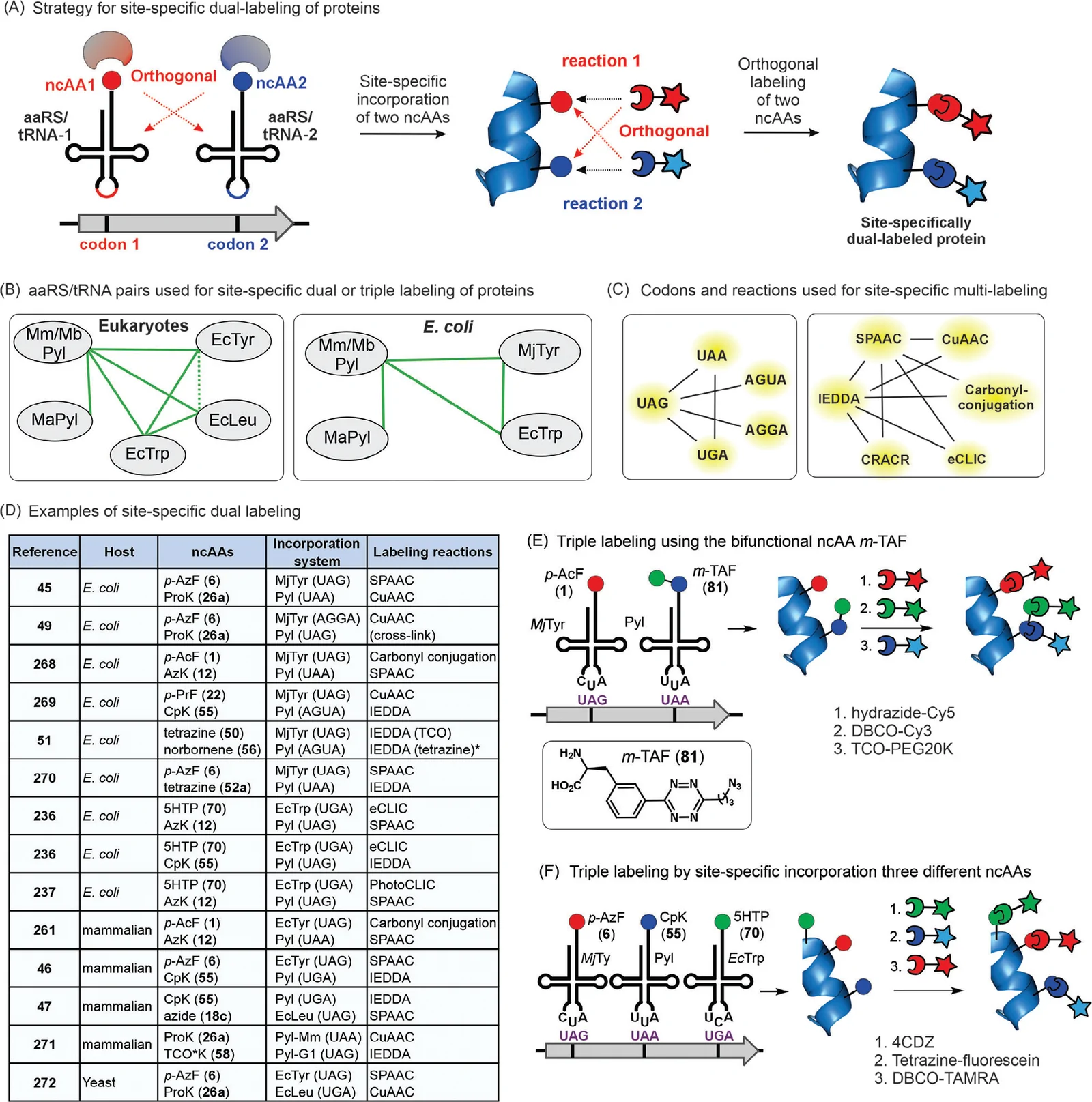
Dual and multiple ncAA-based protein labeling strategies.

**FIGURE 16 |:**
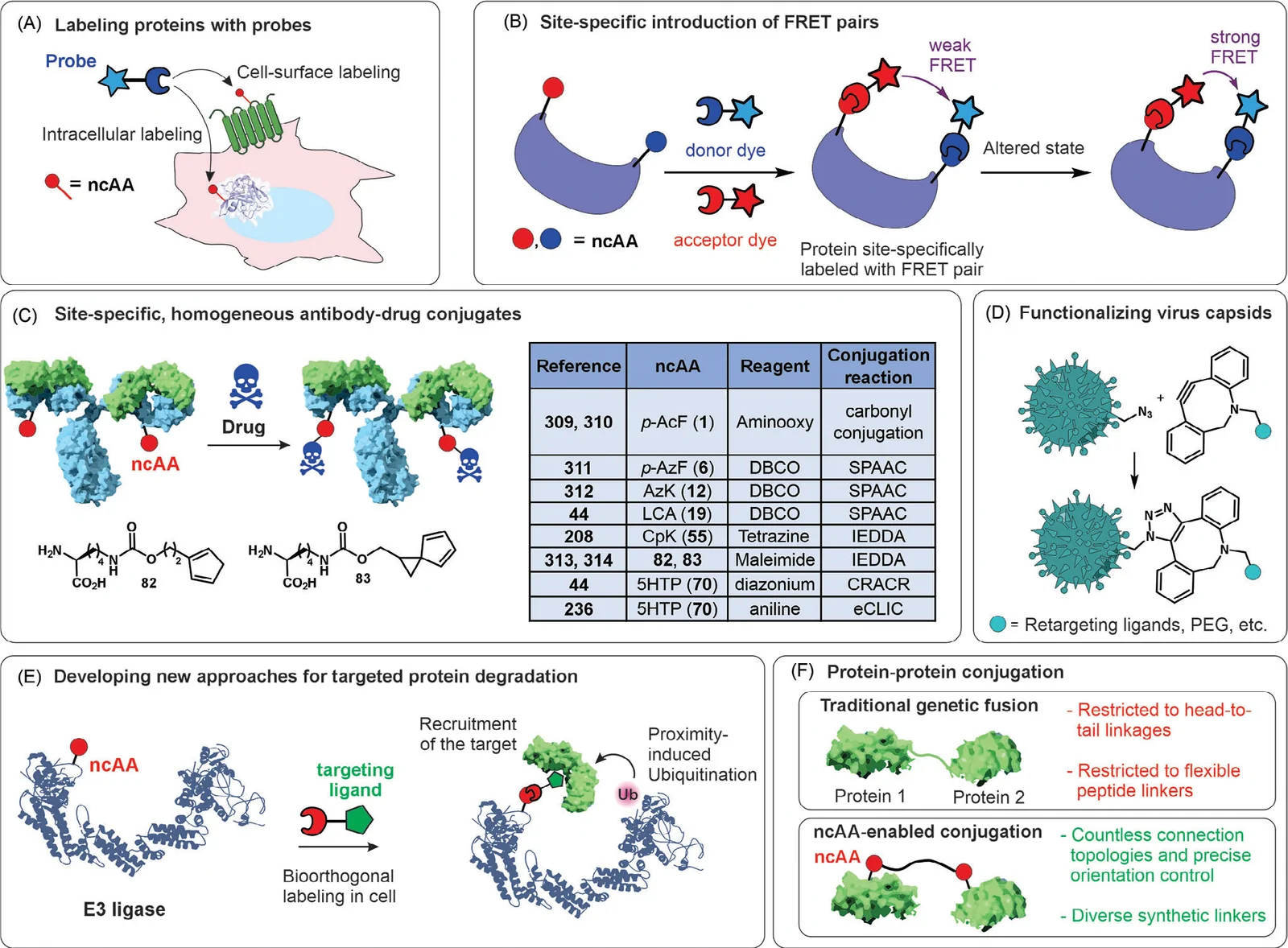
Representative applications of ncAA-enabled protein bioconjugation.

## Data Availability

Data sharing not applicable to this article as no datasets were generated or analyzed during the current study.
